# miR-340-3p-modified bone marrow mesenchymal stem cell-derived exosomes inhibit ferroptosis through METTL3-mediated m^6^A modification of HMOX1 to promote recovery of injured rat uterus

**DOI:** 10.1186/s13287-024-03846-6

**Published:** 2024-07-29

**Authors:** Bang Xiao, Yiqing Zhu, Meng Liu, Meiting Chen, Chao Huang, Dabing Xu, Fang Wang, Shuhan Sun, Jinfeng Huang, Ningxia Sun, Fu Yang

**Affiliations:** 1 Department of Medical Genetics, Naval Medical University, Shanghai, 200433 China; 2 Department of Anatomy, Institute of Biomedical Engineering, Naval Medical University, Shanghai, 200433 China; 3 The Center of Reproductive Medicine, Second Affiliated Hospital of Naval Medical University, Shanghai, 200003 China

**Keywords:** Endometrium injury, miR-340-3p, Exosomes, Ferroptosis, m^6^A modification, METTL3, HMOX1, YTHDF2

## Abstract

**Background:**

Ferroptosis is associated with the pathological progression of hemorrhagic injury and ischemia–reperfusion injury. According to our previous study, exosomes formed through bone marrow mesenchymal stem cells modified with miR-340-3p (MB-exos) can restore damaged endometrium. However, the involvement of ferroptosis in endometrial injury and the effect of MB-exos on ferroptosis remain elusive.

**Methods:**

The endometrial injury rat model was developed. Exosomes were obtained from the supernatants of bone marrow mesenchymal stromal cells (BMSCs) and miR-340/BMSCs through differential centrifugation. We conducted RNA-seq analysis on endometrial tissues obtained from the PBS and MB-exos groups. Ferroptosis was induced in endometrial stromal cells (ESCs) by treating them with erastin or RSL3, followed by treatment with B-exos or MB-exos. We assessed the endometrial total m^6^A modification level after injury and subsequent treatment with B-exos or MB-exos by methylation quantification assay. We performed meRIP-qPCR to analyze m^6^A modification-regulated endogenous mRNAs.

**Results:**

We reveal that MB-exos facilitate the injured endometrium to recover by suppressing ferroptosis in endometrial stromal cells. The injured endometrium showed significantly upregulated *N*^6^-methyladenosine (m^6^A) modification levels; these levels were attenuated by MB-exos through downregulation of the methylase METTL3. Intriguingly, METTL3 downregulation appears to repress ferroptosis by stabilizing HMOX1 mRNA, thereby potentially elucidating the mechanism through which MB-exos inhibit ferroptosis in ESCs. We identified YTHDF2 as a critical m^6^A reader protein that contributes to HMOX1 mRNA degradation. YTHDF2 facilitates HMOX1 mRNA degradation by identifying the m^6^A binding site in the 3′-untranslated regions of HMOX1. In a rat model, treatment with MB-exos ameliorated endometrial injury-induced fibrosis by inhibiting ferroptosis in ESCs. Moreover, METTL3 short hairpin RNA-mediated inhibition of m^6^A modification enhanced the inhibitory effect of MB-exos on ferroptosis in endometrial injury.

**Conclusions:**

Thus, these observations provide new insights regarding the molecular mechanisms responsible for endometrial recovery promotion by MB-exos and highlight m^6^A modification-dependent ferroptosis inhibition as a prospective therapeutic target to attenuate endometrial injury.

**Supplementary Information:**

The online version contains supplementary material available at 10.1186/s13287-024-03846-6.

## Introduction

Intrauterine adhesions (IUAs) refer to an iatrogenic condition characterized by the deterioration of functional endometrium in multiple regions, leading to deterioration of the uterine cavity. This condition develops from aggressive or repeated curettages and/or endometritis, resulting in various complications, including amenorrhea, recurrent pregnancy loss, abnormal placentation, infertility, and psychological distress [1]. IUAs incidence in women with infertility ranges from 2 to 22% [2, 3]. Currently, there is no competent method to prevent IUAs reformation. Surgical treatment with lysis of adhesions through hysteroscopy is the primary therapy for IUAs. However, this approach has shown inconsistent results in alleviating symptoms and improving pregnancy rates [2, 4]. Therefore, alternative approaches are required for treating IUA-related infertility.

Ferroptosis, a distinct cell death pathway, results from iron-dependent oxidation of phospholipids containing polyunsaturated fatty acyl tails [5]. As shown recently, ferroptosis participates in the pathological processes of hemorrhagic injury [6]. Morphological changes in mitochondria, a characteristic of ferroptosis, are observed in nerve cells surrounding hematomas, which provides strong evidence for ferroptosis occurrence after cerebral hemorrhage [7]. Ferroptosis inhibition using ferrostatin 1 is a promising approach in reducing hemorrhagic secondary injury and iron deposition in models of intracerebral hemorrhage [8].

BMSCs contribute to endometrial tissue regeneration because of their immunomodulatory and paracrine functions and their interactions with parenchymal cells [9]. However, BMSCs isolated according current criteria were heterogeneous stromal cells which included stem cells with diverse multipotential properties, committed progenitors, and differentiated cells [10]. Additionally, their clinical application is limited due to heterogeneity and uncertain differentiation ability. Exosomes, which mediate indirect interactions between BMSCs and parenchymal cells by transferring mRNAs, noncoding RNAs, or proteins, have emerged as a potential therapeutic option for IUAs [11]. Exosomes have advantages such as lower immunogenicity than that of BMSCs as well as convenience in administration and storage [12–14]. Modification of exosomes through gene editing in exosome-producing cells has been investigated to enhance their potency and stability [15]. In our previous study on a rat model of uterine injury, BMSCs delivering miR-340 to ESCs through exosomes promoted endometrial regeneration. We also observed that miR-340-modified exosomes derived from BMSCs (MB-exos) significantly reduced injury-induced ferroptosis in ESCs [16]. However, the potential mechanism of MB-exos in ferroptosis in ESCs remains unclear.

*N*^6^-methyladenosine (m^6^A) RNA modification is the most abundant post-transcriptional regulatory mechanism in eukaryotic messenger RNAs (mRNAs) and is involved in numerous biological processes. The dynamic regulation of m^6^A modification is mediated by methyltransferases, demethylases, and m^6^A binding proteins [17]. Recent studies have revealed that exposure to ferroptosis-inducing compounds increases the overall levels of m^6^A modification by upregulating methyltransferase-like 4 and downregulating the demethylase FTO [18]. Furthermore, m^6^A modification appears to activate autophagy by stabilizing BECN1 mRNA, thereby promoting enhanced ferroptosis in hepatic stellate cells [19]. Hence, m^6^A modification plays a crucial role in the regulation of ferroptosis. Exploring the post-transcriptional regulation of m^6^A -mediated ferroptosis in ESCs may offer potential therapeutic targets for preventing IUAs after endometrial injury.

Here, we elucidated the role and molecular mechanisms of ferroptosis for treating endometrial injury through MB-exos administration. Our findings demonstrated that MB-exos treatment reduces the m^6^A methyltransferase METTL3 expression level, consequently enhancing the stability of HMOX1 mRNA by reducing YTHDF2-mediated mRNA degradation, ultimately leading to ferroptosis inhibition in ESCs. Thus, m^6^A modification serves as a novel and critical post-transcriptional ferroptosis regulator in endometrial injury, thereby providing a new strategy to utilize the combination of MB-exos and METTL3 inhibitors for preventing IUAs following endometrial injury.

## Materials and methods

### Animal subjects

Female adult Sprague–Dawley rats (specific pathogen-free; body weight: 200–220 g) were supplied by SLAC Laboratory Animal Co., Ltd. (Shanghai, China). The animals were handled by following the current regulations for animal research [GB14925-2001: Laboratory Animal Requirements of Environment and Housing Facilities (Chinese version)] and received humane treatment. All animal treatments and experimental procedures were granted approval by the Institutional Ethics Committee of the Naval Medical University (approval no. 20190022) and were carried out following the protocol specified in the NIH Guide for the Care and Use of Laboratory Animals.

### Preparation and characterization of miR-340/BMSCs

By using established protocols, we isolated BMSCs from 3-week-old rat bone marrow [16]. The cells were grown in an incubator in low-glucose Dulbecco’s Modified Eagle’s Medium (LG-DMEM; Sigma) containing 10% fetal bovine serum (FBS; Hyclone), 1% penicillin (Sigma), and streptomycin (Sigma) at 37 °C with 5% CO_2_. The third generation of BMSCs was transduced with lentiviruses carrying the LentimiRa-GFP-rno-miR-340 Vector (insertion of pre-miR-340 for knock-in of miR-340, LentiLab) to establish miR-340-modified BMSCs (miR-340/BMSCs). Flow cytometry was conducted to characterize the phenotype of miR-340/BMSCs by using the antibodies (Abs) as follows: anti-CD90, anti-CD44, anti-CD34, and anti-CD29 (all from Abcam) and anti-CD45 (Invitrogen) (Additional file 1: Fig. S1). The miR-340 expression level was confirmed by real-time PCR.

### Isolation and elucidation of exosomes

Exosomes were isolated from the conditioned medium (CM) containing miR-340/BMSCs by using a modified differential ultracentrifugation method [20]. Briefly, miR-340/BMSCs were cultured for 72 h in media containing exosome-free FBS (Sesh-Biotech). After collecting CM, it was centrifuged successively for 10 and 20 min at 300 ×*g* and 3000 ×*g*, respectively, to dispose cell fragments. The CM was further filtered using a filter (0.8 μm, GVS Maine Poretics, Thermo Fisher Scientific) and ultracentrifuged at 4 °C for 2 h at 110,000 ×*g*. Next, the obtained exosomes were enriched at the bottom of the ultracentrifuge tube, resuspended in sterile PBS, and subjected to further centrifugation for 1 h at 100,000 ×*g*. Western blotting (WB) assay was performed to identify exosome markers, including calnexin, CD63, CD9, CD81, Hsp70, and TSG101. Exosome morphology was viewed using a transmission electron microscope (Hitachi; HT 7700). Particle concentration, distribution, and size were quantified by nanoparticle tracking analysis (NTA; Malvern; NS300). The isolated exosomes were freezed at − 80 °C for further analysis.

### Experimental design for the rat endometrial injury model

The endometrial injury rat model was developed in adult female Sprague–Dawley rats based on a previously reported method [21]. Briefly, daily vaginal smears were collected between 4 and 6 PM, and rats showing four consecutive 4-day estrus cycles were chosen for constructing the endometrial injury model. Anesthesia was induced by injecting 3% mebumalnatrium (dose: 1.3 mL/kg) into the lumbar region, followed by a low midline abdominal incision for uterus exposure. A 1.0 cm longitudinal incision was made in the lowermost one-third region of the junction between the uterine distal and middle zones. Subsequently, the uterine wall was scraped until it became coarse, displaying evident endometrial distension.

The animals were assigned to eight groups (6 rats/group at each time point): Sham group, vehicle group, Ferrostatin-1 (Fer-1) group, B-exos (exosomes derived from BMSCs) group, MB-exos group, MB-exos + Lip-control-vector group, MB-exos + Lip-YTHDF2-shRNA group, and MB-exos + Lip-METTL3-shRNA group. In the Sham group, uterine horns were kept intact following a midline incision in the abdomen. In the vehicle group, PBS (500 μL) was administered through caudal vein injection after the injury. The Fer-1 group was intraperitoneally administered 10 mg/kg/d Fer-1 for 2 weeks after surgery. In the MB-exos group, 2.5 × 10^10^ particles of MB-exos in 500 μL PBS were administered through the caudal vein. Within 2 weeks following MB-exos administration, Lip-control-vector or Lip-YTHDF2-shRNA or Lip-METTL3-shRNA (0.75 mg/kg) was intravenously administered thrice per week. After experiment completion, animals in each group were euthanized by inducing anesthesia with 3% mebumalnatrium (dose: 1.3 mL/kg), causing the rats to lose consciousness and then subjected to cervical dislocation. Endometrium samples were obtained from each group and fixed with paraformaldehyde (4% solution; Sigma-Aldrich; P6148) to conduct histopathological examination.

### Hematoxylin and eosin staining

After inducing anesthesia with 3% mebumalnatrium (dose: 1.3 mL/kg), the uterine tissues of rats were collected. Fresh uterine tissues were fixed overnight in paraformaldehyde (4%) at 4 °C and subsequently embedded in paraffin. Subsequently, tissue slices were subjected to staining with hematoxylin and eosin (HE) reagent. A Nikon optical microscope (Tokyo, Japan) was utilized to observe and photograph the slides.

### Masson’s trichrome staining

Masson’s trichrome staining reagent was utilized for staining the uterine tissue sections. Collagen fibers were rendered blue, while the blood vessels, mucosa, muscles, and glands were stained red. Observations and photographic documentation of tissue slices were conducted using an optical microscope. The fibrotic area was quantitatively assessed with ImageJ software (National Institutes of Health, Bethesda, MD, USA).

### Immunohistochemical staining

Formalin-fixed and paraffin-embedded uterine tissue sections from the treated rat model groups were used for immunohistochemical staining. First, tissue slices were deparaffinized with xylene and subjected to rehydration with alcohol. Subsequently, hydrogen peroxide (3% solution) was used for blocking the activity of endogenous peroxidase. Next, retrieval of antigens was achieved through microwave treatment and 0.1 M citric sodium buffer (pH 6.0). The sections were further incubated with primary Abs at 4 °C overnight. An HRP-DAB kit (ZsBio; ZLI-9019) was used for antibody binding detection. Hematoxylin was used for counterstaining. The primary Abs were substituted with the buffer in the negative control group. Images obtained through an Olympus microscope were analyzed with Olympus imaging software.

### Cell senescence β-galactosidase staining

To determine β-gal^ +^ senescent endometrial cells of frozen section in different treatment group, Cell Senescence β-Galactosidase Staining Kit (40754ES60, Beyotime) were used to detect SA-gal activity in cells according to the manufacturer’s instructions.

### Cell experiments

Primary ESCs were isolated from rat endometrium and grown at 37 °C in a humidified atmosphere with 5% CO_2_ using an optimized protocol according previous reported method [22]. The cells were maintained in 10% FBS, 1% penicillin/streptomycin, and 90% DMEM (Gibco; A5669402, 15140163, and 11965092, respectively). ESCs (P_2_-P_5_ generation) were inoculated on the six-well plate at a density of 1 × 10^5^/mL and cultured for 24 h. Then the cells were treated with 2.5 μM RSL3 or 10 μM erastin for ferroptosis induction for 24 h. Concurrently, for the control group, cells exposed to the same volume of DMSO without drugs. In another experiment, ESCs were treated with B-exos or MB-exos (100 μg/mL) for 48 h before and after erastin or RSL3 treatment to assess how B-exos or MB-exos affects ferroptosis. All experiments were repeated thrice.

### Stable cell line construction

Stable cell lines were developed in accordance with a previous protocol [23]. A total of ESCs were seeded onto 6-well plates. After 70% confluence was reached, the cells were added to serum-free DMEM medium. For initiating transfection, the mixture of the plasmid and the transfection reagent (Lipofectamine 3000; Thermo Fisher Scientific; L3000008) was added to each well. Following transfection for 8 h, a serum-free medium was employed to replace 5% FBS-containing medium. Approximately 48 h post-transfection initiation, cell digestion was performed. The cell suspension was then plated in 25 mL culture flasks. Next, we selected a stable cell line by adding puromycin (Thermo Fisher Scientific; A1113803) to the medium at 5 μg/mL concentration for 7 days. WB assay was carried out for estimating transfection efficacy.

### Cell viability assay (CCK8 and EdU)

Cell viability was examined using a Cell Counting Kit 8 (CCK-8) (MCE) by following the supplier’s protocol. A total of 5 × 10^3^ cells were seeded onto 96-well plates; each well was then filled with CCK-8 solution (10 μL). Next, we further incubated the plates for 3 h at 37 °C. Subsequently, absorbance was read with a microplate reader at 450 nm. This absorbance value correlated with viable cell number in the culture.

Cells were placed on the slides of 6-well plates. At approximately 50% confluence, cell proliferation was determined with a Cell-Light EdU Apollo643 in vitro kit (100 T) (Ribobio; C10310-2) in accordance with the supplier’s protocol. After fixing and staining, images of the stained slides were acquired with a microscope.

### Lipid reactive oxygen species assay

Flow cytometry with the dye BODIPY-C11 (Thermo Fisher; D3861) was conducted to estimate lipid reactive oxygen species (ROS) levels. Cells were seeded onto six-well plates (density: 2–3 × 10^5^ cells/well). The cells were grown for 12 h and subjected to B-exos or MB-exos treatment after pretreatment for 24 h with DMSO, RSL3, or erastin. The culture medium was substituted by complete medium (2 mL) containing BODIPY-C11 (5 µM). The cells were incubated further for 20 min. After cell harvesting in 15 mL tubes, cells were subjected to washing with PBS twice for eliminating excess BODIPY-C11 and resuspended in 300 µL PBS. Next, a nylon mesh (0.4 µm) cell strainer was used to filter the cell suspension; flow cytometry was then conducted to detect cell lipid ROS level based on the shift in the fluorescence emission peak (590–510 nm); the result was equivalent to lipid ROS generated. A total of 50,000 cells per condition were analyzed.

### Iron assay

Intracellular ferrous iron (Fe^2+^) levels were quantified with the iron assay kit (Abcam; ab83366) in accordance with the supplier’s protocol. Cells (density: 5 × 10^6^ cells/10 cm plate) were subjected to B-exos or MB-exos treatment for 24 h before and after pretreatment with DMSO, RSL3, or erastin. After cell collection, cells were subjected to ice-cold PBS washing. This was followed by homogenization of the cells on ice using iron assay buffer (5 × volume). Insoluble substances were removed by centrifuging the homogenates at 4 °C at 13,000 ×*g* for 10 min. After supernatant collection, each sample was supplemented with an iron reducing agent, followed by mixing and incubation at room temperature (RT) for 30 min. This was followed by the addition of an iron probe (100 μL) to each sample, thorough mixing, and incubation in the dark for 60 min at RT. A microplate reader was utilized for measuring absorbance at 593 nm.

### Malondialdehyde assay

The relative malondialdehyde (MDA) levels in cell lysates were quantified with the lipid peroxidation assay kit (Abcam; ab118970) in accordance with the supplier’s protocol. Cells (density: 5 × 10^6^ cells/10 cm plate) were subjected to B-exos or MB-exos treatment for 24 h before and after pretreatment with DMSO, RSL3, or erastin. Cells or uterine tissue (10 mg) were subjected to ice-cold PBS washing thrice, followed by homogenization in MDA lysis buffer (300 μL) with 100 × BHT (3 μL) on ice. Insoluble substances were removed by centrifuging the homogenates at 4 °C at 13,000 ×*g* for 10 min. From each homogenized sample, 200 μL of the supernatant was added to a microcentrifuge tube; TBA solution (600 μL) was then added. This mixture was placed at 95 °C for 60 min to allow reaction between MDA from the sample and TBA, leading to the formation of the MDA–TBA adduct. After cooling the samples to RT for 10 min on an ice bath, a 200 μL aliquot was transferred to a 96-well plate. Subsequently, absorbance at 532 nm was measured using a microplate reader.

### Glutathione assay

We utilized the glutathione assay kit (Sigma; CS0260) for quantifying relative glutathione (GSH) levels in uterus tissues or cell lysates in accordance with the supplier’s instructions. For cell experiments, cells (density: 5 × 10^6^ cells/10 cm plate) were subjected to B-exos or MB-exos treatment for 24 h before and after pretreatment with DMSO, RSL3, or erastin. After washing thrice with ice-cold PBS, the cells were lysed with lysis buffer (1%) containing Tris (25 mM, pH 7.5), EDTA (1 mM), NP-40 (0.5%), and NaCl (300 mM) together with a phosphatase inhibitor mix (Pierce) and a complete protease inhibitor cocktail (Roche). The lysate was sonicated (Thermo Model 120) and subsequently centrifuged for 10 min at 13,200 rpm at 4 °C. The cleared lysate was utilized for assessing GSH level in each sample. Enzymes that could interfere with the analysis were eliminated using a deproteinizing sample kit (ab204708). This was followed by the addition of a GSH assay mixture (50 µL) to each reaction well and 60-min incubation at RT in the dark environment. GSH measurement was performed through a kinetic assay wherein the catalytic amount of GSH continuously reduced 5,5′-dithiobis (2-nitrobenzoic acid) to 5-thio-2-nitrobenzoic acid, and the reaction rate indicated the GSH level. 5-Thio-2-nitrobenzoic acid (yellow product) was quantified by measuring absorbance at 412 nm using a spectrophotometer.

### Oxidized glutathione assay

The relative levels of oxidized glutathione (GSSG) in uterus tissues or cell lysates were determined with a kit (Cayman; 703002) by following the supplier’s instructions. For cell experiments, cells (density: 5 × 10^6^ cells/10 cm plate) were subjected to B-exos or MB-exos treatment for 24 h before and after pretreatment with DMSO, RSL3, or erastin. Following washing thrice with ice-cold PBS, the cells were collected using a rubber policeman. After homogenization with cold 50 mM MES buffer (2 mL), insoluble substances were removed by centrifuging the homogenate at 4 °C for 15 min at 10,000 ×*g*. GSH was selectively removed through derivatization using 2-vinylpyridine (Sigma; 132292) to quantify GSSG. The supernatant was collected, and 2-vinylpyridine solution (10 μL, 1 M in ethanol) was added per milliliter of sample. Following vortexing, the mixture was incubated at RT for 60 min for blocking the thiol groups already present in GSH. Next, 5 μL of glutathione reductase (2 U/mL) and 95 μL of NADPH (2 mg/mL) in water were added for reducing GSSG. Subsequently, the sample (50 μL/well) was placed in a 96-well plate, and the assay cocktail mixture (150 μL) was prepared and added to each well containing the samples. The 96-well plate was incubated on a shaker in a dark environment for 30 min. The absorbance reading was quantified at 412 nm with a microplate reader.

### Transmission electron microscopy

Morphological features of ESCs undergoing ferroptosis were assessed by transmission electron microscopy (TEM), as reported previously [24]. Briefly, we seeded and cultured ESCs (20,000 cells/well) on a cover glass with a 4-well chamber (Thermo Fisher Scientific; 155382). Images were obtained with a transmission electron microscope (Olympus, EM208S).

### RNA extraction and RT-qPCR

TRIzol reagent (Sangon Biotech; B610409-0100) was utilized to isolate total RNA from cells or uterus tissues as mentioned in the supplier’s manual. RNA purity and concentration were quantified with a Nanodrop spectrophotometer. The Hifair® III 1st Strand cDNA Synthesis SuperMix for qPCR (Yeasen; 11141ES60) was utilized for cDNA synthesis from the isolated RNA. By using the Hieff® qPCR SYBR Green Master Mix (Yeasen; 11202ES50), qRT-PCR was carried out on an ABI 7500 system (Applied Biosystems). We utilized the 2^−∆∆Ct^ method for estimating fold change, and GAPDH was used as an internal reference. Additional file 2: Table S1 provides primer sequence details.

### Western blot assay

For western blot assay, ESCs were lysed in RIPA buffer (0.1% SDS, Tris pH 7.4 [50 mM], 0.5% sodium deoxycholate, 150 mM NaCl, 1% NP-40) augmented with a protease inhibitor (Roche; 5892791001). The BCA protein assay kit (Sangon Biotech; C503021-0500) was utilized for estimating total protein level in the supernatant. Proteins were subjected to PAGE for separation and then transferred to PVDF membranes. Subsequently, the membranes were blocked for 30 min using 5% skimmed milk and incubated at 4 ℃ overnight with the appropriate primary Abs. The membranes were further incubated for 1 h with secondary Abs at RT. A chemiluminescence system (Bio-Rad, Hercules, CA) was utilized to visualize the protein bands. All full-length WB images are provided in Additional file 3.

### Construction of the HMOX1 mutant plasmid

HMOX1 mutants were constructed with PCR-based methods as reported previously [25]. The CDS, 3′-UTR, and 5′-UTR regions of HMOX1 mRNA were synthesized and subsequently subcloned into the pCDNA3.1 vector. Adenine (A) 1193 and 1236 were mutated to guanine (G). DNA sequencing was conducted to confirm HMOX1 wild-type (WT) and mutants.

### Luciferase reporter assay

To determine rat HMOX1 activity, the CDS, 3′-UTR, and 5′-UTR regions were cloned into the pGL3-Luc vector (Promega, E1751) [24]. Subsequently, the ESCs were transfected with (1) pGL3-Luc-HMOX1-3′UTR, (2) pGL3-Luc-HMOX1-CDS, and (3) pGL3-Luc-HMOX1-5′UTR plasmids. Additionally, the transfection efficiency was monitored by co-transfection of the internal monitor phRL-null (Promega, E2231) with the luciferase reporter vectors.

### Quantification of m^6^A RNA methylation

The EpiQuik™ kit (Colorimetric) to quantify m^6^A RNA methylation was utilized for quantifying m^6^A RNA methylation. First, based on the supplier’s protocol, TRIzol reagent (Sangon Biotech, B610409-0100) was utilized for isolating total RNA from uterus tissues. m^6^A was recognized with detection Abs and specific capture m^6^A Abs. The detected signal was amplified and estimated with a colorimetric method by reading absorbance at 450 nm with a microplate spectrophotometer. m^6^A amount was equivalent to OD intensity measured.

### MeRIP-qPCR

m^6^A immunoprecipitation (MeRIP) qPCR was performed as reported previously [26]. Briefly, 5 μg of m^6^A Abs and normal rabbit IgG were conjugated overnight at 4℃ to Protein A/G Plus Agarose (50 μL). The Abs were kept overnight at 4℃ with fragmented total RNA (100 μg) in an immunoprecipitation buffer (750 mM NaCl, 50 mM Tris–HCl, and 0.5% NP40) containing 40 U RNase inhibitor (Beyotime; R0102). To elute RNAs, we incubated the beads with an elution buffer (300 μL, 1 mM EDTA, 5 mM Tris–HCl, and 0.05% SDS), supplemented with proteinase K (8.4 μg; ST535, Beyotime) at 50℃ for 1.5 h. Subsequently, following TRIzol and ethanol precipitation, reverse transcription of the input and m^6^A-enriched RNAs was performed, and real-time PCR was utilized to analyze enrichment degree. Additional file 2: Table S2 shows the primer sequences for MeRIP-qPCR.

### RIP-RT-qPCR

RNA immunoprecipitation (RIP) was carried out with a previously reported protocol [27]. Briefly, by using a Stratalinker, ESCs were irradiated two times (wavelength: 254 nm; dose: 400 mJ/cm^2^). Subsequently, following a wash with cold PBS, a high-salt lysis buffer (Tris–HCl [pH 7.6, 20 mM], 0.5 mM DTT, 0.2% NP-40, 300 mM NaCl, protease inhibitor, and RNase inhibitor [200 U/mL]) was used to lyse ESCs at 4℃ for 30 min. Furthermore, we incubated the supernatant with anti-YTHDF2 Abs in 1 × IP buffer (500 μL) containing RNase inhibitors or Protein A/G Plus agarose (Santa Cruz Biotechnology, sc-2003) overnight at 4℃ following RNase T1 (1 U; Thermo Fisher Scientific, EN0541) treatment for 15 min at 24 ℃. Elution buffer (100 μL) containing 0.05% SDS, Tris–HCl (5 mM, pH 7.5), 20 mg/mL Proteinase K, and EDTA (1 mM, pH 8.0) was then added and incubated at 50 ℃ for 2 h. Finally, RNAs related to YTHDF2 were recovered through precipitation with TRIzol and ethanol; the recovered RNAs were quantified by real-time PCR.

### RNA sequencing

TRIzol reagent (Invitrogen, 15596018) was utilized for total RNA extraction from ESCs or endometrial tissues. For each sample, a library was prepared using 1 μg of RNA with the TruSeq Stranded Total RNA kit and Ribo-Zero™ magnetic gold kit (Epicentre; MRZG126). Reads of all samples were aligned with those of the rat reference genome with TopHat v1.4.1. We utilized EdgeR version 3.08 to estimate differential gene expression. Furthermore, the Benjamini-Hochburg approach was employed for calculating adjusted P values. Downregulation and upregulation were considered at fold change < 0.65 and > 1.4, respectively.

### Immunofluorescence assay

For immunofluorescence staining, ESCs (density: 2 × 10^4^ cells/well) were seeded onto 24-well plates and treated for specified time periods with the indicated compounds. Subsequently, ESCs were treated with anti-HMOX1 primary Abs (Invitrogen; MA1-112) overnight at 4℃. Next, FITC-labeled secondary Abs (Thermo Fisher; A16079) were added. DAPI (Sigma-Aldrich; MBD0015) was used for nuclear counterstaining. An inverted fluorescence microscope (Axio Observer, Zeiss) was utilized to acquire the images.

### Statistical analysis

A large sample size as well as multiple independent repeats carried out by independent investigators were used to ensure sufficient statistical power and reduce estimation error. Multiple experiments with different quantification methods yielded mutually supportive and consistent findings. The sample size was determined based on our extensive research experience and literature review. Mean ± SD was used for expressing data. The mean values between two groups were compared with unpaired Student’s t-test. Furthermore, multiple groups were compared with one-way analysis of variance (ANOVA); a post hoc test with the least significant difference test was utilized for significant findings in ANOVA. GraphPad Prism 6.0 (GraphPad Software, Inc.) was utilized for evaluating data. Statistical significance was considered at *P* < 0.05.

### ARRIVE checklist

All authors declare that the work has been reported in line with the ARRIVE guidelines 2.0.

## Results

### MB-exos promote healing of the injured rat uterus and inhibit cell senescence

**Fig. 1:**
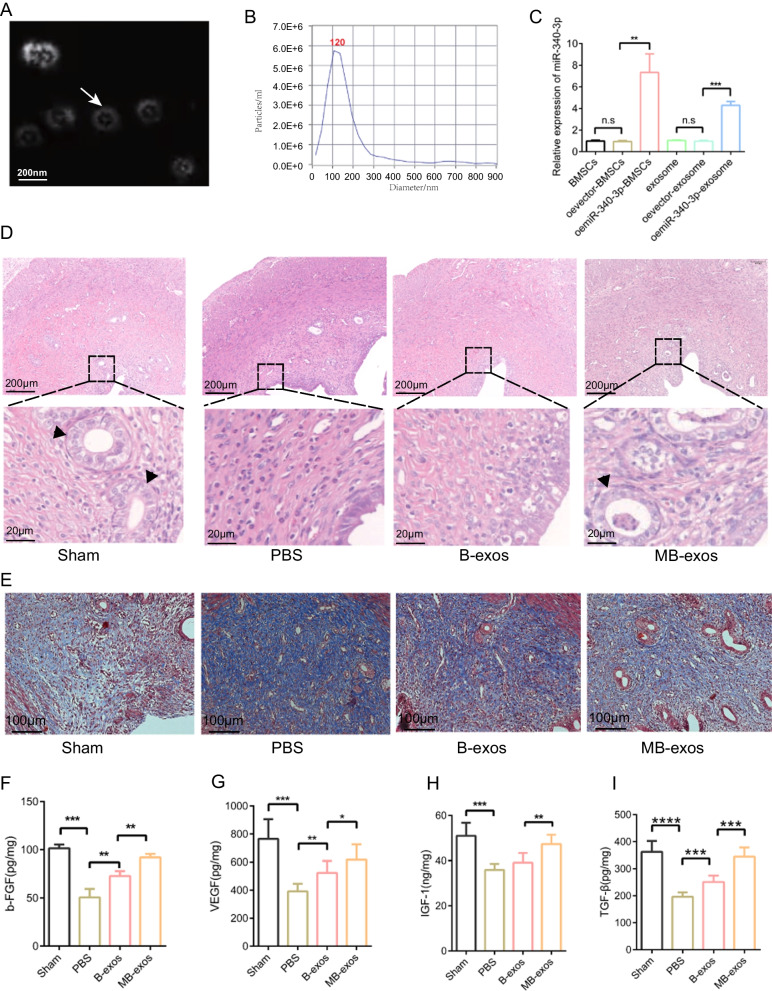
MB-exos promote injured uterus recovery. **A** Transmission electron microscopy images showing exosome morphology, characterized by a standard bilayer membrane structure. **B** Exosome particle size analysis. **C** RT-qPCR analyzed miR-340-3p expression in BMSCs, oemiR-340-3p-BMSCs, and exosomes derived from them [n.s.: not significant, **P < 0.01, ***P < 0.001] (n = 3/group). **D** Representative images of hematoxylin and eosin-stained uterus tissues from the Sham, PBS, B-exos, and MB-exos groups (n = 6/group). **E** Representative images of Masson’s trichrome-stained uterus tissues from the Sham, PBS, B-exos, and MB-exos groups (n = 6/group). **F**–**I** b-FGF, VEGF, IGF-1, and TGF-β levels in uterine tissue extracts from each group [*P < 0.05, **P < 0.01, ***P < 0.001, ****P < 0.0001] (n = 6/group). Abbreviations: BMSCs: bone marrow mesenchymal stem cells; B-exos: BMSC-derived exosomes; MB-exos: miR-340-3p-modified BMSCs-derived exosomes

Exosomes were obtained from the supernatants of BMSCs and miR-340/BMSCs through differential centrifugation. TEM analysis confirmed the presence of exosomes with typical bilayer membrane vesicles (Fig. 1A). NTA revealed a mean particle size of 123.6 ± 3.2 nm for the exosomes (Fig. 1B), with 1.1 × 10^11^ particles/mL concentration. Western blot assay revealed that exosomal surface markers, including HSP70, CD63, TSG101, CD9, and CD81, were expressed in exosomes; however, calnexin was absent (Additional file 1: Fig. S2). Furthermore, miR-340-3p levels were notably elevated in both miR-340-3p-exosomes and miR-340/BMSCs compared to BMSCs and vector-exosomes, respectively (Fig. 1C). By using SD rats as a model, we successfully established an endometrium injury model (Additional file 1: Fig. S3A). HE staining was performed to evaluate histological morphology of the injured uterus. The sham group exhibited normal endometrial morphology, with glands present in the basal layer and submucosa and minimal collagen accumulation (Fig. 1D). Compared to the PBS group, increased endometrial thickness was observed in both B-exos-treated and MB-exos-treated groups (Fig. 1D, Additional file 1: Fig. S3B). The endometrial fibrosis extent was determined by Masson staining. Treatment with B-exos or MB-exos significantly decreased the percentage of fibrotic area in the endometrium (Fig. 1E, Additional file 1: Fig. S3C). The expression levels of growth factors, including insulin-like growth factor 1 (IGF-1), vascular endothelial growth factor (VEGF), basic fibroblast growth factor (bFGF), and transforming growth factor β (TGF-β), in the injured uterus were estimated by ELISA (Fig. 1F-I). The bFGF, VEGF, IGF-1, and TGF-β levels were higher in the B-exos and MB-exos groups than in the PBS group. Compared to B-exos treatment, MB-exos treatment further increased endometrial thickness, reduced fibrosis levels, and upregulated the expression of growth factors. Besides, cell senescence plays a vital role in various physiological and pathological contexts, including tissue repair processes. Therefore, we detected the expression of cell senescence marker β-galactosidase and P21 in different treatment groups (Additional file 1: Fig. S4A and B), and found that B-exos and MB-exos down-regulated the expression of damage-induced β-galactosidase and P21, and MB-exos had stronger inhibitory capacity than B-exos. This suggests that MB-exos can significantly inhibit cell senescence caused by injury. Thus, stem cell-derived exosomes have a beneficial effect for repairing the injured endometrium, with MB-exos demonstrating superior therapeutic efficacy as compared to B-exos.

### Ferroptosis activation diminishes the beneficial effect of MB-exos or B-exos

**Fig. 2:**
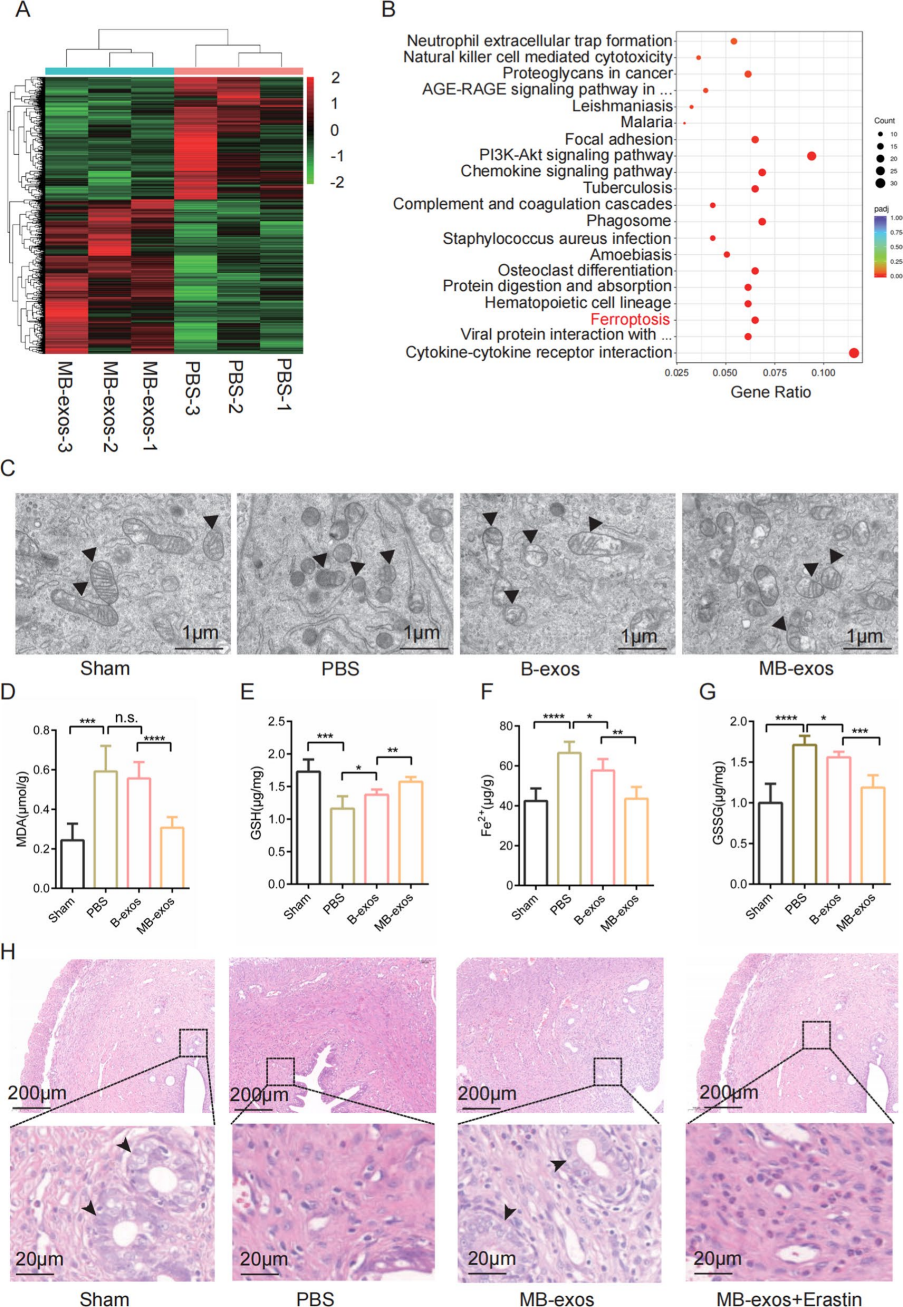
MB-exos promote recovery of the injured uterus through the inhibition of ferroptosis. **A** Heatmap displaying the DEGs between the PBS and MB-exos groups (n = 3). **B** DEGs were evaluated with KEGG analysis, and 20 significantly enriched pathways are presented. **C** Transmission electron microscopy images showing mitochondrial morphology in the endometrium of different groups (Sham, PBS, B-exos, and MB-exos) (n = 3/group). **D**–**G** Assay results for MDA production, GSH depletion, iron accumulation, and GSSG levels in each group (Sham, PBS, B-exos, and MB-exos) [n.s.: not significant, *P < 0.05, **P < 0.01, ***P < 0.001, ****P < 0.0001] (n = 6/group). **H** Representative images of hematoxylin and eosin (HE)-stained uterus tissues from the Sham, PBS, MB-exos, and MB-exos + erastin groups (n = 6/group)

To reveal the potential mechanisms through which MB-exos facilitate endometrial recovery post-injury, we conducted RNA-seq analysis on endometrial tissues obtained from the PBS and MB-exos groups. Differentially expressed genes (DEGs) were identified between these groups; their relative expression levels are shown in the heatmap presented in Fig. 2A. Subsequently, we carried out Kyoto Encyclopedia of Genes and Genomes (KEGG) and Gene Ontology (GO) analyses for understanding biological processes and signaling pathways influenced by these DEGs. Figure 2B illustrates the top 20 signaling pathways, with a notable emphasis on ferroptosis, which is a known consequence of iron overload and oxidative stress associated with hemorrhagic injury [8]. Experimental validation was performed to substantiate the findings of the enrichment analysis. TEM analysis revealed an intact mitochondrial bilayer structure of endometrial cells, with clearly visible mitochondrial ridges in the sham group. Compared to PBS treatment, B-exos treatment significantly restored the mitochondrial bilayer membrane structure, exhibiting characteristics associated with ferroptosis, such as concentration, membrane thickening, reduction or absence of ridges, and injury-induced outer membrane rupture. Notably, MB-exos displayed a considerably higher capacity to restore the mitochondrial bilayer membrane structure than B-exos treatment (Fig. 2C). Lipid peroxidation, iron overload, GSH depletion, and lipid ROS accumulation are the distinctive characteristics of ferroptotic cells. We conducted assays on adjacent endometrial tissues to assess the levels of ferroptosis markers, including MDA, GSH, Fe^2+^, and GSSG (Figs. 2D-G). Moreover, compared to PBS treatment, B-exos treatment caused a significant reduction in MDA, Fe^2+^, and GSSG levels, while increasing GSH levels. Additionally, compared to B-exos treatment, MB-exos significantly enhanced the effects of B-exos. The administration of the ferroptosis activator erastin counteracted the effects of MB-exos, including the increased endometrial thickness; increased bFGF, VEGF, and IGF-1 levels (Fig. 2H, Additional file 1: Fig. S5C–F); and decreased fibrosis area following injury (Additional file 1: Fig. S5A, B). Thus, ferroptosis activation can offset the beneficial effects of MB-exos on the injured uterus.

### MB-exos exert inhibitory effects on ferroptosis in ESCs

**Fig. 3:**
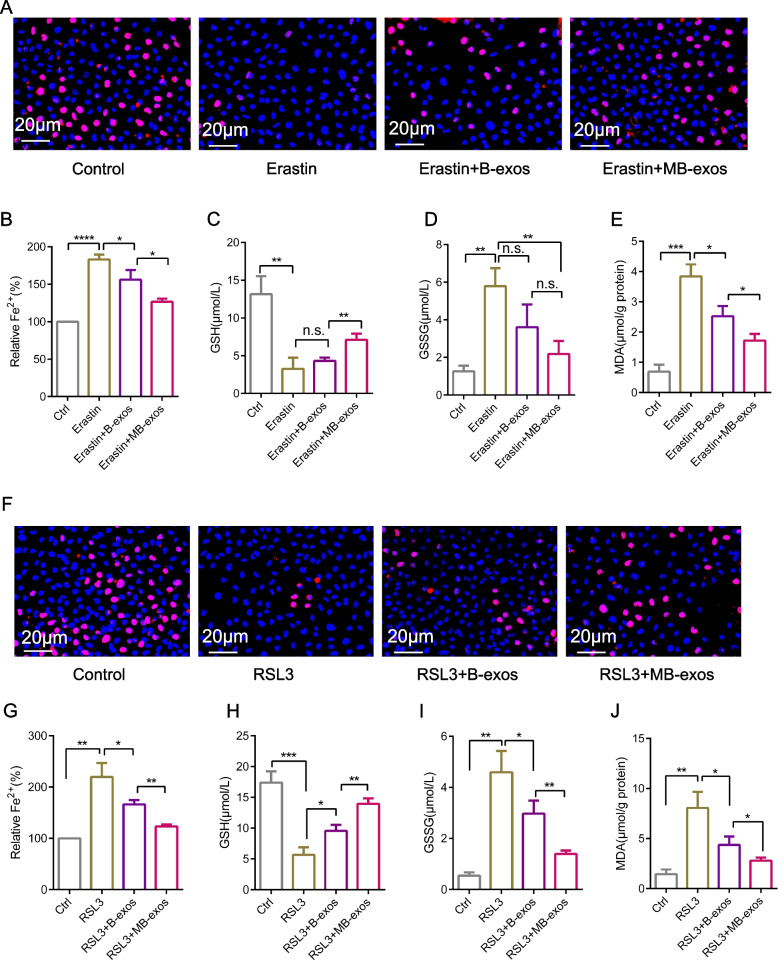
MB-exos inhibit RSL3- or erastin-induced ferroptosis of endometrial stromal cells. **A** ESC cell proliferation was examined by EdU staining after erastin (10 μM) treatment with/without B-exos or MB-exos (n = 3/group). **B**–**E** Iron accumulation, GSSG levels, MDA production, and GSH depletion were measured in each group (control, erastin, B-exos + erastin, and MB-exos + erastin) (n = 3/group) [n.s.: not significant, *P < 0.05, **P < 0.01, ***P < 0.001, ****P < 0.0001]. ESCs were subjected to RSL3 (2.5 μM) treatment with/without B-exos or MB-exos. (**F**) Cell proliferation was evaluated by EdU staining (n = 3/group). **G**–**J** Iron accumulation, GSSG levels, GSH depletion, and MDA production were measured in each group (control, RSL3, B-exos + RSL3, and MB-exos + RSL3) [*P < 0.05, **P < 0.01, ***P < 0.001] (n = 3/group)

To assess how MB-exos affects ferroptosis in endometrial cells, ferroptosis was induced in ESCs by treating them with erastin or RSL3, followed by treatment with B-exos or MB-exos. Treatment with erastin or RSL3, the ferroptosis activator, decreased cell viability significantly, while B-exos or MB-exos restored cell viability. MB-exos exhibited a stronger restorative effect on cell viability than B-exos following treatment with erastin (Fig. 3A, Additional file 1: Fig. S6B) or RSL3 (Fig. 3F, Additional file 1: Fig. S6C). Additionally, erastin or RSL3 exposure significantly increased cellular lipid ROS levels (Additional file 1: Fig. S5D–G), Fe^2+^ levels (Fig. 3B and G), GSSG levels (Figs. 3D and I), and MDA levels (Fig. 3E and J) and decreased GSH levels (Fig. 3C and H). Conversely, B-exos or MB-exos treatment significantly offset erastin or RSL3 effects on the levels of these markers. The MB-exos group exhibited a greater reduction in cellular ferroptosis than the B-exos group, thus indicating a stronger inhibitory effect of MB-exos on ferroptosis.

### m^6^A modification is implicated in injury-induced ferroptosis in ESCs

**Fig. 4:**
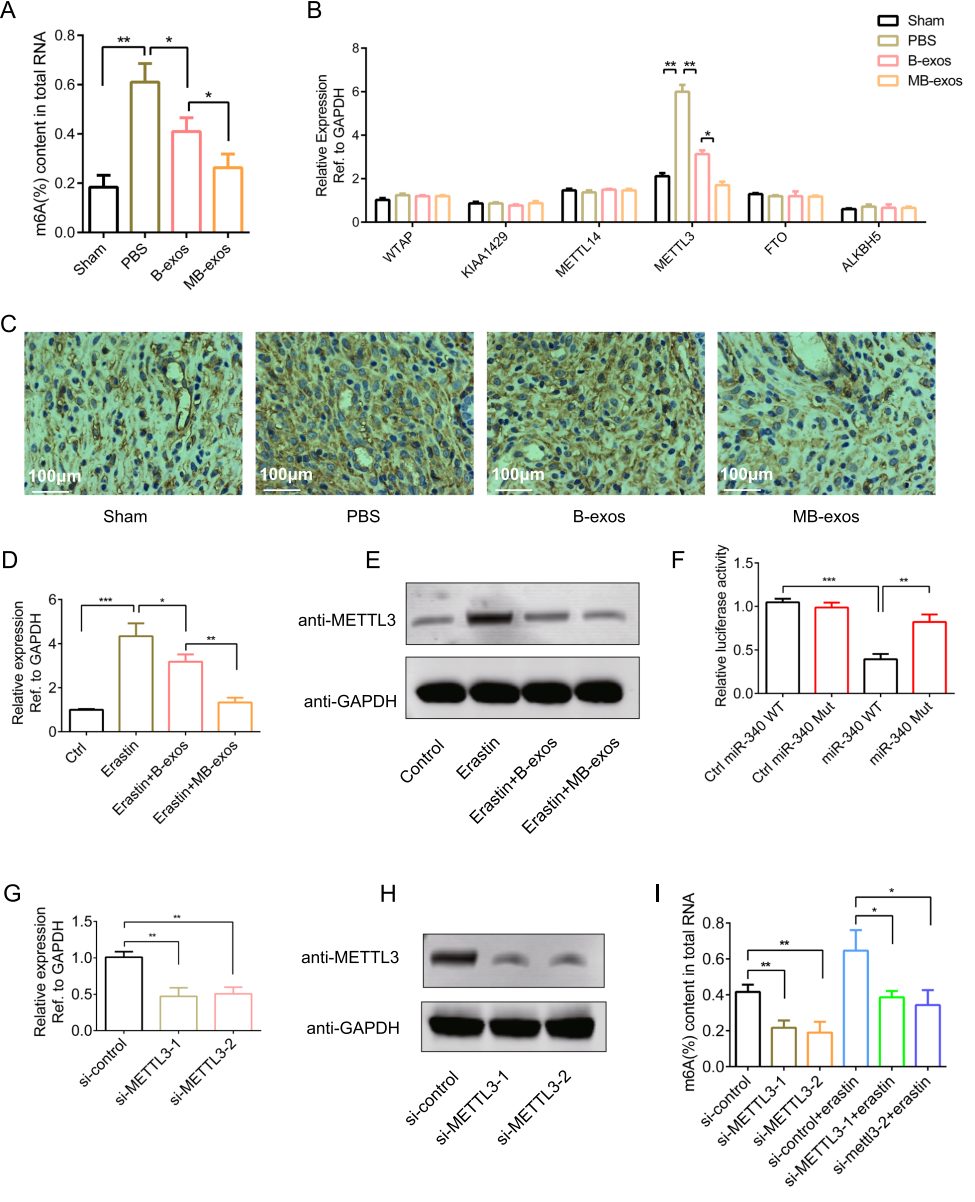
m^6^A modification downregulation is mediated by METTL3 after treatment with MB-exos. **A** The m^6^A RNA Methylation Quantitative kit was utilized for detecting endometrial m^6^A modification in the different groups (Sham, PBS, B-exos, and MB-exos) [*P < 0.05, **P < 0.01] (n = 3/group). **B** mRNA levels of m^6^A writers in the Sham, PBS, B-exos, and MB-exos groups were measured by RT-qPCR [*P < 0.05, **P < 0.01] (n = 3/group). **C** METTL3 expression in different groups (Sham, PBS, B-exos, and MB-exos) was assessed by immunohistochemistry (n = 3/group). ESCs were subjected to erastin (10 μM) treatment with B-exos or MB-exos for 24 h. RT-qPCR **D** as well as western blotting assay **E** were carried out for measuring METTL3 mRNA and protein expression levels [*P < 0.05, **P < 0.01, ***P < 0.001] (n = 3/group). **F** Dual luciferase reporter gene assay showed the targeted binding of miR-340-3p to the 3′-UTR of METTL3 mRNA in ESCs [**P < 0.01, ***P < 0.001] (n = 3/group). **G**, **H** METTL3 expression was knocked down using siRNA [**P < 0.01] (n = 3/group). **I** m^6^A levels were detected in ESCs transfected with METTL3 siRNA followed by 24-h erastin treatment (10 μM) [*P < 0.05, **P < 0.01] (n = 3/group)

m^6^A modification can regulate various cellular processes, including necroptosis, apoptosis, endothelial-mesenchymal transition (EMT), and senescence [17]. However, its involvement in regulating injury-induced ferroptosis in ESCs remains unclear. To investigate this possibility, we assessed the endometrial total m^6^A modification level after injury and subsequent treatment with B-exos or MB-exos. The m^6^A RNA methylation quantification assay revealed a remarkable increase in m^6^A modification levels following injury. B-exos or MB-exos treatment markedly decreased the m^6^A modification level compared with that after PBS treatment. Notably, MB-exos treatment showed a stronger capacity to downregulate m^6^A modification levels (Fig. 4A). Furthermore, compared to PBS treatment, treatment with B-exos or MB-exos significantly downregulated the METTL3 mRNA expression level in comparison with that of other methyltransferases. METTL3 expression was more prominently downregulated by MB-exos treatment than by B-exos treatment (Fig. 4B). The results of immunohistochemical staining for METTL3 expression in the endometrium corroborated these findings (Fig. 4C).

At the cellular level, we observed that erastin upregulated protein and mRNA expression levels of METTL3, which was downregulated significantly by B-exos treatment. MB-exos treatment demonstrated a stronger inhibitory effect on METTL3 expression than B-exos treatment (Fig. 4D and E). The dual luciferase reporter assay revealed METTL3 as a target of miR-340-3p (Fig. 4F). To investigate whether the upregulation of m^6^A modification directly contributes to ferroptosis induction, we transfected ESCs with METTL3 siRNA (Fig. 4G and H). The m^6^A RNA methylation quantification assay confirmed that METTL3 knockdown completely abrogated the erastin-induced upregulation of m^6^A modification in ESCs (Fig. 4I).

### Inhibition of m^6^A modification confers resistance to ferroptosis in ESCs

**Fig. 5:**
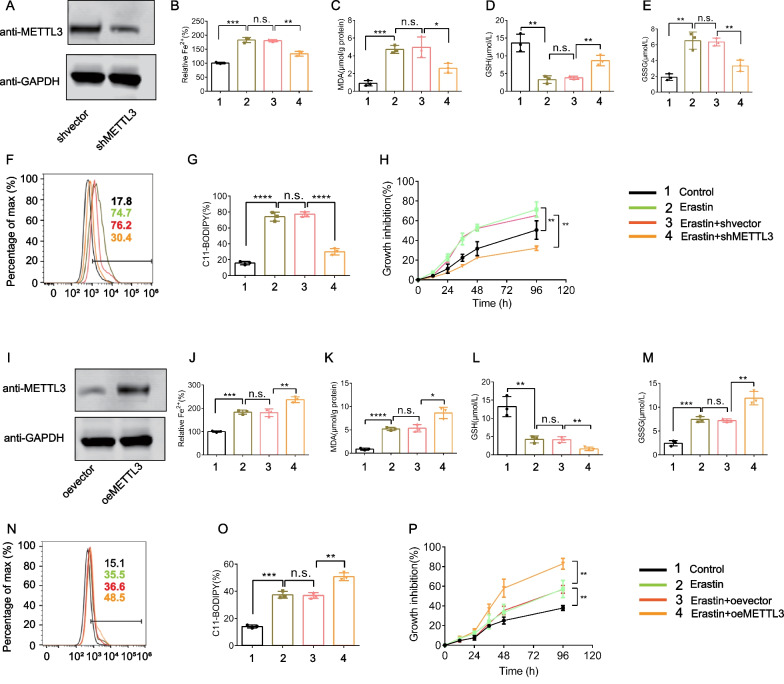
METTL3 regulates erastin-induced ferroptosis of ESCs. METTL3 knockdown suppressed erastin-induced ferroptosis (**A**–**H**). METTL3 shRNA was stably transfected into ESCs, followed by 24-h erastin treatment (10 μM). **A** METTL3 expression was quantified by immunoblotting and compared with GAPDH levels (n = 3/group). **B** Fe^2+^ accumulation was estimated by an iron detection assay [n.s.: not significant, **P < 0.01, ***P < 0.001] (n = 3/group). **C** The lipid formation was measured by MDA assay [n.s.: not significant, *P < 0.05, ***P < 0.001] (n = 3/group). GSH (**D**) and GSSG **E** levels were estimated by relative assay kits [n.s.: not significant, **P < 0.01] (n = 3/group). **F**, **G** Flow cytometry with C11-BODIPY was carried out for estimating the lipid ROS level [n.s.: not significant, ****P < 0.0001] (n = 3/group). **H** Cell viability was estimated with the Cell Counting Kit-8 [**P < 0.01] (n = 3/group). Overexpression of METTL3 enhanced ferroptosis induced by erastin (**I**–**P**). **I** Immunoblotting was carried out to estimate METTL3 expression levels, which were compared with GAPDH levels (n = 3/group). METTL3 plasmid was stably transfected into ESCs, followed by 24-h erastin treatment (10 μM). **J** Fe^2+^ accumulation was quantified by an iron detection assay [n.s.: not significant, **P < 0.01, ***P < 0.001] (n = 3/group). **K** Lipid formation was quantified by MDA assay [*P < 0.05, ****P < 0.0001, n.s.: not significant] (n = 3/group). GSH (**L**) and GSSG **M** levels were quantified by relative assay kits [**P < 0.01, ***P < 0.001, n.s.: not significant] (n = 3/group). **N**, **O** Flow cytometry with C11-BODIPY was carried out to quantify the lipid ROS level [n.s.: not significant, **P < 0.01, ***P < 0.001] (n = 3/group). **P** Cell viability was evaluated by the Cell Counting Kit-8 kit [**P < 0.01] (n = 3/group)

To confirm the involvement of m^6^A modification in ferroptosis regulation, we transfected ESCs with METTL3 shRNA (Fig. 5A) or plasmid (Fig. 5I) to establish stable cell clones with METTL3 knockdown or overexpression, respectively. The results showed that METTL3 shRNA significantly abolished growth inhibition induced by erastin and RSL3 treatment in ESCs (Fig. 5H, Additional file 1: Fig. S7G), while the METTL3 plasmid enhanced the inhibition of ESCs growth (Fig. 5P, Additional file 1: Fig. S7N). m^6^A modification inhibition by METTL3 shRNA clearly impaired the erastin- or RSL3-induced accumulation of redox-active iron (Fig. 5B, Additional file 1: Fig. S7A), lipid ROS generation (Fig. 5F and G, Additional file 1: Fig. S7E and F), GSH depletion (Fig. 5D, Additional file 1: Fig. S7C), and GSSG production (Fig. 5E, Additional file 1: Fig. S7D) and MDA (Fig. 5C, Additional file 1: Fig. S7B). Conversely, METTL3 overexpression enhanced the effect of erastin or RSL3 in inducing ferroptosis in ESCs (Fig. 5J–P, Additional file 1: Fig. S7H–N). Thus, m^6^A modification regulation by METTL3 may be associated with ferroptosis in ESCs.

### METTL3 downregulation inhibits ferroptosis in ESCs by regulating m.^6^A modification in HMOX1

**Fig. 6:**
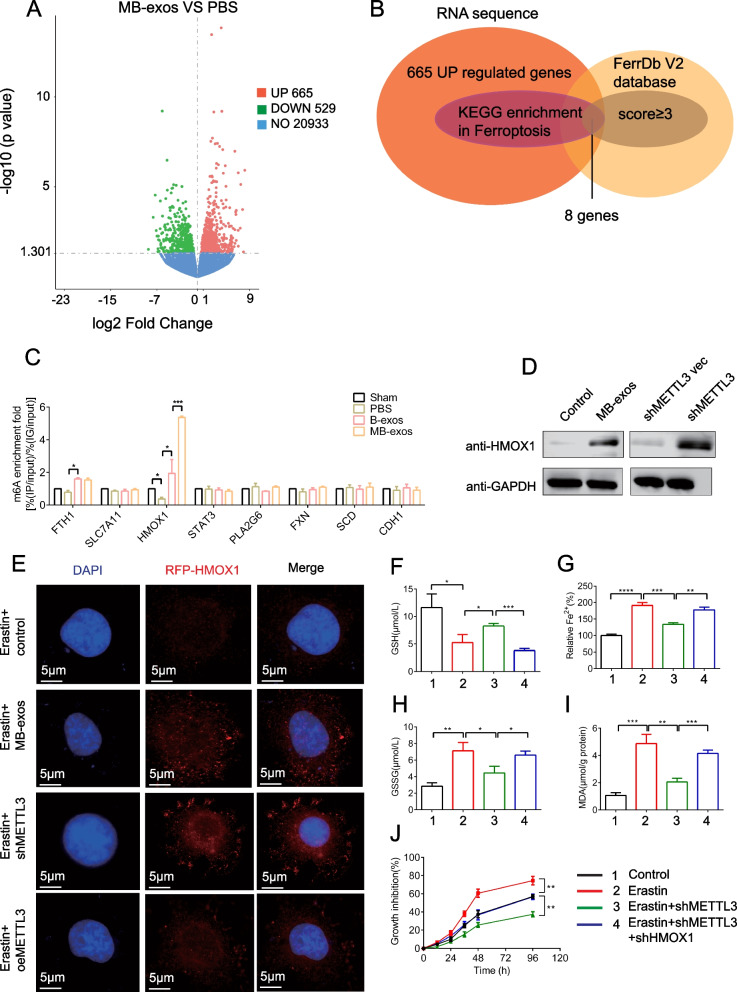
METTL3 regulates ferroptosis of ESCs through the regulation of m6A modification of HMOX1. Rats were subjected to PBS, B-exos, and MB-exos treatment for 3 days following endometrium injury. **A** Volcano plot showing the DEGs in the MB-exos and PBS groups (n = 3). **B** RNA sequencing and FerrDb V2 database screening for target genes of METTL3 (screening process). **C** m6A modification levels in candidate genes of the endometrium in various groups were quantified by MeRIP-qPCR [*P < 0.05, **P < 0.01] (n = 3/group). **D** ESCs were pretreated with MB-exos or transfected with METTL3 shRNA plasmid and then subjected to 24-h erastin treatment (10 μM). Western blotting assay was conducted for evaluating HMOX1 protein expression (n = 3/group). **E** MB-exos, METTL3 shRNA, or METTL3 plasmid was transferred into ESCs followed by 24-h erastin treatment (10 μM). Immunofluorescence was performed for detecting HMOX1 expression. Representative photographs are shown. Scale bars: 5 μm. HMOX1 shRNA and METTL3 shRNA were transfected into ESCs and subjected to 24-h erastin treatment (10 μM). GSH (**F**), Fe^2+^ (**G**), GSSG (**H**), and MDA **I** concentrations were estimated by relative assay kits [*P < 0.05, **P < 0.01, ***, *P* < 0.001] (n = 3/group). **J** Cell viability was determined by the Cell Counting Kit-8 kit (**P < 0.01; n = 3/group)

To elucidate the molecular mechanism underlying ferroptosis regulation by m^6^A modification in ESCs, we combined RNA-seq results and previously reported ferroptosis-related genes in the FerrDb V2 database to screen the involved target genes (Fig. 6A and B). The screening process is shown in Fig. 6B, and 8 differential genes were screened. Subsequently, we performed meRIP-qPCR to analyze m^6^A modification-regulated endogenous mRNAs (Fig. 6C). The m^6^A modification levels in heme oxygenase 1 (HMOX1), a critical target gene known to inhibit ferroptosis, were upregulated by B-exos or MB-exos treatment. Notably, compared to B-exos treatment, MB-exos treatment exhibited a significantly stronger upregulation of m^6^A modification in HMOX1 (Fig. 6C). Consistent with these findings, the reduction in the m^6^A modification level by MB-exos or METTL3 shRNA markedly increased HMOX1 protein expression (Fig. 6D). Immunofluorescence assay of ESCs further confirmed that MB-exos or METTL3 shRNA significantly upregulated HMOX1 expression during ferroptosis in ESCs, while METTL3 overexpression down-regulated it (Fig. 6E).

To assess whether HMOX1 upregulation mediated the resistance to ferroptosis induced by m^6^A modification inhibition, we utilized HMOX1 shRNA for activating ferroptosis. As expected, the findings showed that METTL3 shRNA impaired growth inhibition induced by erastin, while the HMOX1 shRNA intensely enhanced growth inhibition during ferroptosis in ESCs (Fig. 6J). METTL3 shRNA pretreatment completely abolished lipid ROS accumulation (Additional file 1: Fig. S8A), iron accumulation, MDA production, GSSG production, and GSH depletion (Fig. 6G, I, H, and F, respectively) in ESCs treated with erastin. However, the inhibition of m^6^A modification did not abolish ferroptotic events significantly in HMOX1 shRNA presence (Fig. 6F–J). Additionally, TEM assays further confirmed that ferroptosis reduction following the downregulation of m^6^A modification by METTL3 was rescued by HMOX1 shRNA in ESCs (Additional file 1: Fig. S8B). Thus, HMOX1 knockdown impairs the resistance of m^6^A modification inhibition to ferroptosis in ESCs.

### YTHDF2, the m^6^A reader protein, enhances HMOX1 mRNA degradation by identifying the m^6^A binding site

**Fig. 7:**
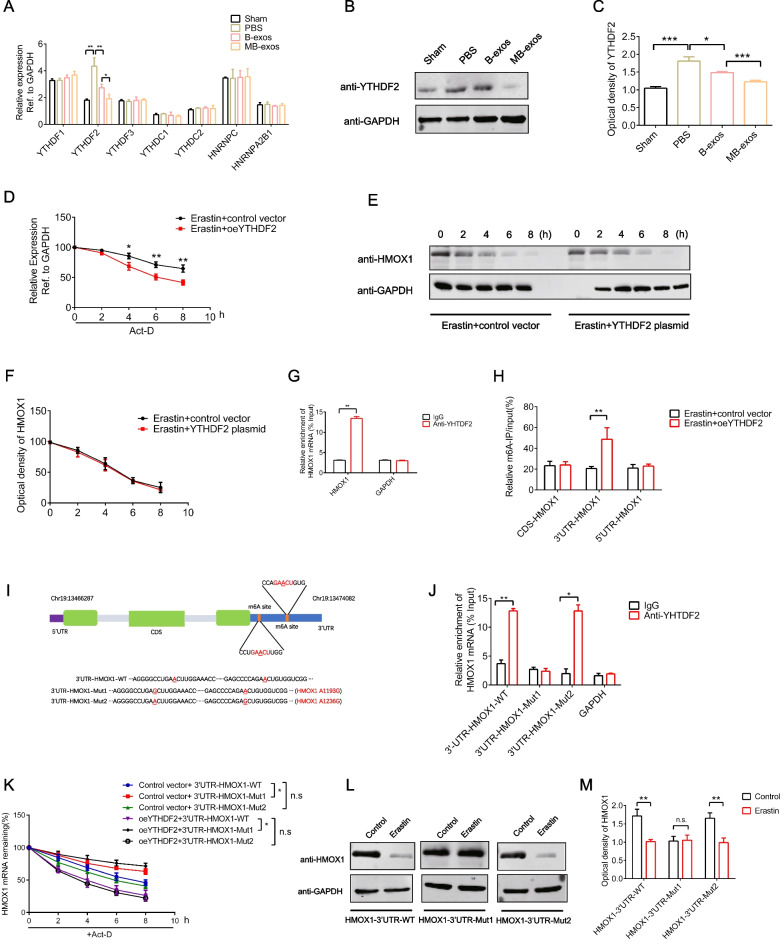
YTHDF2 (m^6^A reader protein) enhances HMOX1 mRNA degradation by identifying the m^6^A binding site. Rat endometrium was treated with B-exos, MB-exos, or PBS for 3 days following injury. **A** mRNA levels of m^6^A readers were quantified by RT-qPCR [*P < 0.05, **P < 0.01] (n = 3/group). **B** Western blotting assay was carried out for estimating the protein level of the m6A reader YTHDF2 (n = 3/group). **C** The optical density of YTHDF2 in all groups was analyzed (*P < 0.05, **P < 0.001). **D** ESCs were transfected with the control vector or the YTHDF2 plasmid and subjected to 24-h treatment with 10 μM erastin, followed by 5 μg/mL Act-D exposure for specific durations. RT-qPCR was conducted for quantifying the remaining HMOX1 mRNA levels [*P < 0.05, **P < 0.01] (n = 3/group). **E** ESCs were transfected with the control vector or the YTHDF2 plasmid and subjected to 24-h erastin treatment (10 μM). Western blotting assay was carried out for estimating HMOX1 protein levels at various time points under CHX (100 μg/mL) treatment. **F** The optical density of HMOX1 at all time points in the two groups was analyzed (n = 3/group). **G** YTHDF2 and HMOX1 mRNA binding was estimated by RIP (n = 3/group) [*P < 0.01]. **H** ESCs were transfected with the control vector or the YTHDF2 plasmid and then subjected to 24-h erastin treatment (10 μM). m^6^A modification levels in HMOX1 mRNA CDS, 5′-UTR, and 3′-UTR were estimated by MeRIP-qPCR [**P < 0.01] (n = 3/group). **I** Schematic representation of the position and mutation of m^6^A motif in 3′-UTR in HMOX1 mRNA is shown. **J** The binding of YTHDF2 with HMOX1-3′UTR-WT, HMOX1-3′UTR-Mut1 (A1193G) and HMOX1-3′UTR-Mut2 (A1236G) in ESCs was assessed by YTHDF2 RIP (n = 3/group) [*P < 0.05, **P < 0.01]. **K** ESCs were transfected with the control vector or the YTHDF2 plasmid, followed by transfection with HMOX1-3′UTR-WT, HMOX1-3′UTR-Mut1, or HMOX1-3′UTR-Mut2 plasmid; ESCs were subjected to pretreatment with Act-D (5 μg/mL) for specific durations. RT-qPCR was carried out for determining the remaining HMOX1 mRNA [n.s.: not significant, *P < 0.05] (n = 3/group). **L** ESCs were subjected to 10 μM erastin treatment for 48 h following transfection with HMOX1-3′UTR-WT, HMOX1-3′UTR-Mut1, or HMOX1-3′UTR-Mut2 plasmid. Western blotting assay was utilized to estimate HMOX1 protein expression. **M** The optical density of HMOX1 in each group was analyzed [n.s.: not significant, **P < 0.01] (n = 3/group)

m^6^A modification in ferroptosis is controlled by specific m^6^A reader proteins. To determine which m^6^A reader protein participates in METTL3-dependent m^6^A modification-mediated ferroptosis regulation, we conducted an unbiased screening among m^6^A reader proteins. Interestingly, our results revealed that YTHDF2, among other m^6^A readers, was significantly upregulated after endometrial injury and was significantly downregulated by B-exos treatment. Furthermore, MB-exos treatment resulted in a considerably stronger downregulation of YTHDF2 expression compared to B-exos treatment (Fig. 7A–C). YTHDF2 can recognize m^6^A-methylated mRNA and participates in the post-transcriptional control of its target genes. Growing evidence suggests that YTHDF2 binds to m^6^A sites on transcripts, thereby promoting mRNA degradation [28]. In our study, as shown in Fig. 7D, HMOX1 mRNA’s half-life was reduced in ESCs transfected with the YTHDF2 plasmid compared to that in the control group. Consistently, no significant difference was noted in the half-life of HMOX1 between the YTHDF2 overexpression and control groups in ESCs treated with the translation inhibitor cycloheximide (Fig. 7E and F). To confirm the binding of YTHDF2 to HMOX1 mRNA in ESCs, we performed RNA–protein immunoprecipitation (RIP). As depicted in Fig. 7G, the HMOX1 PCR products displayed a high enrichment in YTHDF2 samples in comparison with the reference PCR product, thus indicating a direct binding of YTHDF2 to HMOX1 mRNA. MeRIP-qPCR revealed that m^6^A modification was enriched significantly in the HMOX1 3′-UTR region (Fig. 7H). A detailed sequence analysis showed a conserved 5-nucleotide consensus sequence (GAACU) in the HMOX1 mRNA 3′-UTR sequence (Fig. 7I, Additional file 1: Fig. S9A). To analyze how m^6^A -binding sites affect the HMOX1 3′-UTR region, we generated HMOX1-3′-UTR-WT, HMOX1-3′-UTR-Mut1 (A1193G), and HMOX1-3′-UTR-Mut2 (A1236G) constructs (Fig. 7I). According to RNP IP assays, HMOX1-3′-UTR-Mut1, but not HMOX1-3′-UTR-Mut2, impaired YTHDF2 binding to HMOX1 mRNA (Fig. 7J). Consistently, in RNA stability assays, the YTHDF2 plasmid shortened the half-life of HMOX1-3′-UTR-WT mRNA and HMOX1-3′-UTR-Mut2, but not HMOX1-3′-UTR-Mut1 mRNA (Fig. 7K). Additionally, the results of WB assay indicated that HMOX1-3′-UTR-Mut1 significantly compromised erastin-induced downregulation of the HMOX1 protein (Fig. 7L and M). Thus, YTHDF2 overexpression promotes HMOX1 mRNA degradation through binding at the m^6^A binding site in the 3′-UTR region, specifically at position A1193.

### Inhibition of m^6^A modification impairs injury-induced ferroptosis in the rat endometrium

**Fig. 8:**
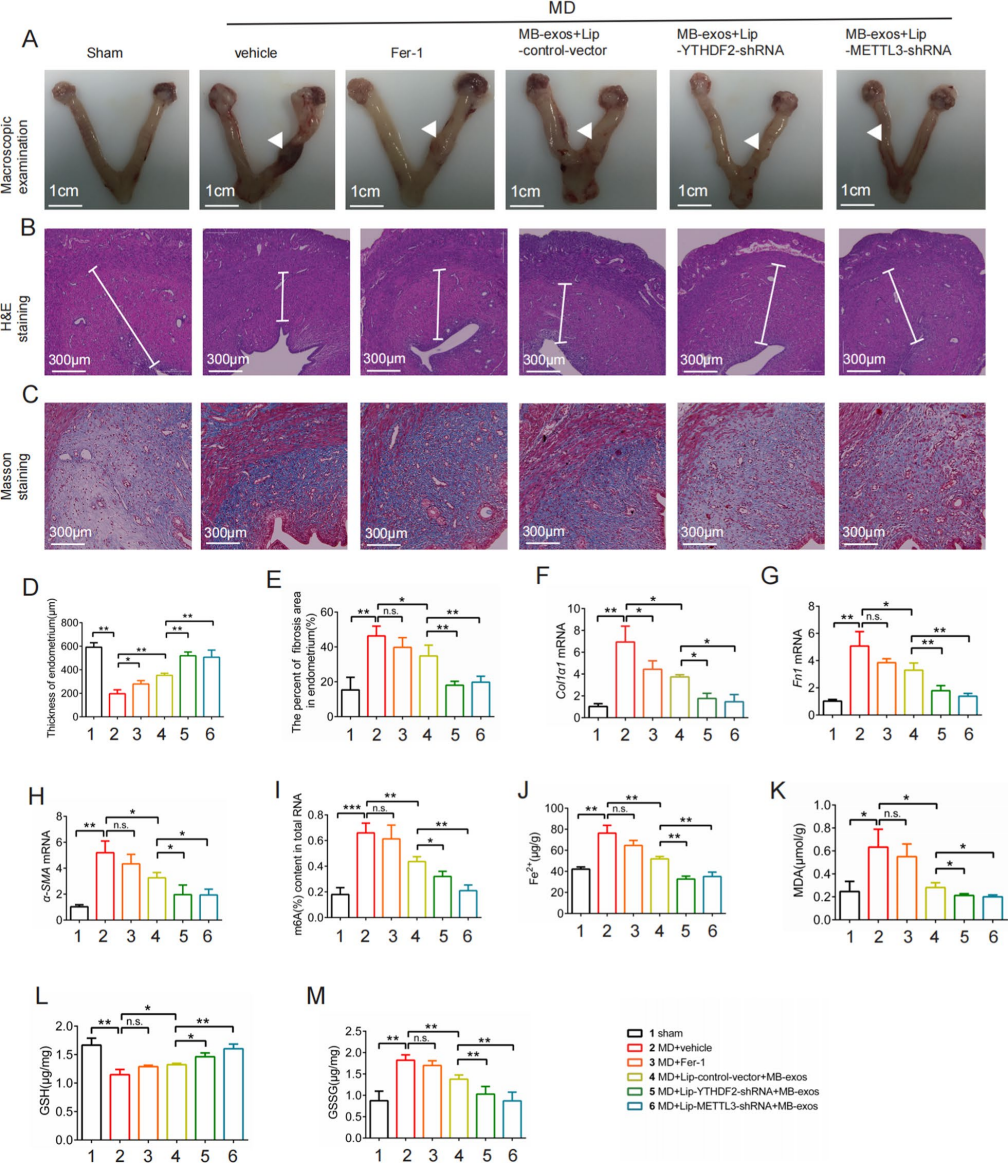
m^6^A modification inhibition impairs injury-induced ferroptosis in the rat endometrium. Rats were divided into 6 groups: Sham, MD + Vehicle, MD + Fer-1, MD + Lip-control-vector + MB-exos, MD + Lip-YTHDF2-shRNA + MB-exos, and MD + Lip-METTL3-shRNA + MB-exos. **A** Macroscopic examination was carried out for observing pathological changes in the uterus; the arrow indicates the injury site. Scale bars: 1 cm. HE staining (**B**) and Masson staining **C** were employed for histopathological analysis. Representative images are shown. Scale bars: 100 μm. Statistics of endometrial thickness (**D**) and the fibrotic area percentage **E** in the endometrium of each group [n.s., not significant, *P < 0.05, **P < 0.01] (n = 3/group). **F–H** mRNA expression levels of endometrial fibrosis markers (Fn1, α-SMA, and Col1a1) were estimated by real-time PCR [n.s.: not significant, *P < 0.05, **P < 0.01] (n = 3/group). **I** The m^6^A levels were quantified with the m^6^A RNA methylation quantitative kit [n.s.: not significant, *P < 0.05, **P < 0.01, ***P < 0.001] (n = 3/group). Accumulation of iron (**J**), production of MDA (**K**), depletion of GSH (**L**), and production of GSSG **M** were assessed [n.s.: not significant, *P < 0.05, **P < 0.01] (n = 3/group)

To assess how the inhibition of m^6^A modification affects ferroptosis and the subsequent recovery of the injured endometrium in vivo, we utilized liposomes carrying METTL3 shRNA (Lip-METTL3-shRNA) or YTHDF2 shRNA (Lip-YTHDF2-shRNA) in combination with MB-exos to treat the injured rat uteri. Macroscopic examination revealed that the injury model group exhibited pathological changes compared to the control group, while MB-exos treatment alleviated injury-induced endometrium fibrosis (Fig. 8A). HE staining demonstrated that MB-exos treatment significantly restored the injured uterus’s morphological structure (Fig. 8B). Masson staining showed that the fibrotic pathology observed in the model group of endometrium fibrosis was mitigated by MB-exos treatment (Fig. 8C). Interestingly, pretreatment with Lip-METTL3-shRNA or Lip-YTHDF2-shRNA greatly enhanced the therapeutic effects of MB-exos on endometrium fibrosis (Fig. 8C and E). The results also indicated that Lip-METTL3-shRNA or Lip-YTHDF2-shRNA significantly augmented the inhibitory effect of MB-exos on Col1a1, Fn1, and α-SMA mRNA expression levels (Fig. 8F–H). To clarify how m^6^A modification influences injury-induced ferroptosis in ESCs, m^6^A levels were determined with an m^6^A RNA methylation quantitative kit. Lip-METTL3-shRNA or Lip-YTHDF2-shRNA significantly enhanced the inhibition of m^6^A modification by MB-exos (Fig. 8I). Moreover, the inhibition of m^6^A modification by Lip-METTL3-shRNA or Lip-YTHDF2-shRNA greatly potentiated the inhibitory effect of MB-exos on injury-induced ferroptosis in ESCs, as evidenced by reduced redox-active iron levels, MDA and GSSG elimination, and increased GSH levels (Fig. 8J–M). These observations support the assumption that m^6^A modification inhibition can enhance the inhibitory function of MB-exos on injury-induced ferroptosis in ESCs, thereby enabling the injured rat uterus to recover.

## Discussion

The incomplete repair of the endometrium after injury can be attributed to the absence of normal cells or impaired cell function. BMSCs participate in the functional recovery of the endometrium by interacting with uterine parenchymal cells and promoting ESCs proliferation. Our previous study revealed that exosomes derived from BMSCs inhibited fibrosis in injured ESCs through the mediation of miR-340, although the underlying mechanism remained unclear. Here, we identified that exosomes derived from miR-340-3p modified BMSCs exerted an inhibitory effect on ferroptosis by modulating METTL3-mediated m^6^A modification of HMOX1, thereby facilitating the recovery of the wounded rat uterus.

Exosomes serve as a crucial vehicle for the biological functions of MSCs and offer advantages over cell therapy by avoiding potential complications [29]. Modifying MSCs is an effective approach to maximize the therapeutic properties of exosomes derived from MSCs. For instance, according to Zheng et al., hemin pretreatment increased miR-183-5p content in MSC-derived exosomes, leading to an enhanced inhibitory effect on cardiomyocyte senescence [30]. In addition, exosomal miR-9-5p derived from iPSC-MSCs ameliorates doxorubicin-induced cardiomyopathy by inhibiting cardiomyocyte senescence [31]. Because the pathological process of IUAs is highly complex, there is still no clear and effective clinical treatment strategy. Exosomes have emerged as an ideal vector to deliver therapeutic molecules. At the same time, increasing numbers of studies have shown that miRNAs play a momentous role in the process of IUAs [32]. In our study, MB-exos exhibited a more pronounced ability to promote the recovery of the wounded uterus as compared to B-exos, thus indicating that miR-340-3p level modulation improved the therapeutic efficacy of exosomes derived from BMSCs. All these results suggest that exosome-loaded miRNAs may be a potentially effective treatment for endometrial injury.

Ferroptosis, a recently discovered controlled cell death program, is triggered through iron-dependent lipid peroxidation and represents a distinct type of cell death compared to other well-known forms [6, 33, 34]. There is mounting evidence implicating ferroptosis in the pathological processes of ischemia–reperfusion injury and traumatic injury [35]. Neuronal ferroptosis is confirmed in in vitro and in vivo models of intracerebral hemorrhage; moreover, ferroptosis inhibition has shown a promising result in reducing injury caused by intracerebral hemorrhage. Consistent with previous research, our study demonstrated that injury-induced ferroptosis could be attenuated by B-exos or MB-exos, resulting in increased cell viability and alleviation of endometrium fibrosis. Notably, MB-exos exhibited a stronger inhibitory effect on ferroptosis than B-exos, possibly due to the increased levels of miR-340-3p, a known regulator of multiple genes. Recognizing the involvement of ferroptosis in endometrium fibrosis development after injury provides novel insights for diagnosis and treatment. However, the precise role of ferroptosis in inhibiting endometrium fibrosis by MB-exos remains undefined, and there are important considerations and limitations that should be addressed.

m^6^A is the main internal mRNA modification that occurs in the consensus motif RRACH of eukaryotic organisms [36, 37]. m^6^A motifs are primarily enriched in the CDS and 3ʹ-UTR regions and are critically involved in managing precursor mRNA alternative splicing, degradation, and translation. Based on confirmation by extensive investigations, m^6^A modification is associated with the progression and occurrence of various diseases, thus highlighting its participation in monitoring cell fate, including senescence, necroptosis, and apoptosis [38–40]. Notably, according to Wang et al., m^6^A modification promotes adipogenesis by activating autophagy [41]. Additionally, Ma et al. noted the upregulation of the m^6^A methylase METTL14 in human hepatocellular carcinoma, and silencing METTL14 inhibits hepatocellular carcinoma cell proliferation significantly and promotes apoptosis [42]. The functional role and the mechanism underlying m^6^A modification in ferroptosis are, however, unclear. Here, we found an increase in m^6^A modification in the endometrium after injury, mediated by the upregulation of the methylase METTL3 during ferroptosis in ESCs. Importantly, MB-exos reduced m^6^A modification by downregulating METTL3, resulting in the inhibition of ferroptosis in ESCs after injury. Although further investigations are necessary for determining the precise role of m^6^A modification in the inhibition of ferroptosis in ESCs by MB-exos, our findings reveal a novel function of MB-exos in controlling m^6^A modification.

RNA-binding proteins carrying the YTH domain, such as YTHDF1/2/3 and YTHDC1/2, are the m^6^A motif readers. They identify and then bind to m^6^A-modified transcripts, thereby controlling gene expression [43, 44]. Numerous studies have demonstrated that YTHDF2 specifically recognizes m^6^A-methylated mRNAs and facilitates their post-transcriptional regulation [45–48]. For instance, Yu et al. found that YTHDF2 identifies m^6^A-modified TP53 and PER1 mRNAs, thereby promoting their degradation and accelerating ocular melanoma tumorigenesis [49]. In hepatocellular carcinoma, YTHDF2 modulates m^6^A methylation for OCT4 mRNAs, promoting cancer metastasis and cancer stem cell liver phenotype [50]. Additionally, according to Hou et al., YTHDF2 SUMOylation enhances its binding affinity for m^6^A -modified mRNAs, thereby causing gene expression dysregulation and cancer progression [51]. Nevertheless, the precise role of YTHDF2 in regulating other tissues or cell types remains to be elucidated, as it may exhibit distinct effects on specific m^6^A modifications during disease occurrence and development. In our present study, YTHDF2 was found to promote HMOX1 mRNA degradation by identifying the m^6^A binding site in the HMOX1 3′UTR region at position A1193. Disrupting the m^6^A sites in HMOX1 abrogated YTHDF2-mediated mRNA degradation, thereby facilitating ferroptosis in ESCs. Our findings offer a new perspective regarding the potential underlying mechanism of ferroptosis regulation by YTHDF2.

## Conclusion

**Fig. 9:**
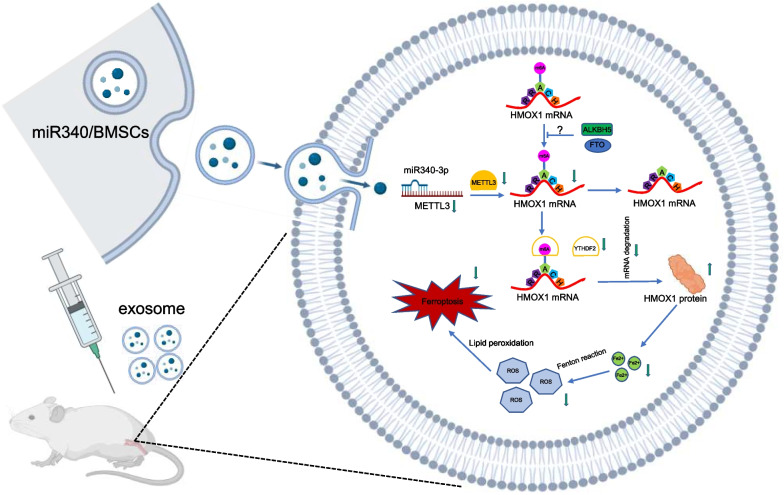
Inhibition of m^6^A modification impairs ESCs ferroptosis by MB-exos. The downregulation of methylase METTL3 decreased the levels of m^6^A modifications in HMOX1 mRNA. The downregulation of m^6^A reader YTHDF2 decreased HMOX1 mRNA degradation via recognizing the m^6^A binding site, thus inhibiting ferroptosis in ESCs and eventually leading to the recovery of the injured uterus

To summarize, we demonstrated the involvement of ferroptosis in the endometrial repair process after injury. MB-exos were found to inhibit ESCs ferroptosis by downregulating METTL3 expression, thereby decreasing HMOX1 degradation and promoting injured rat uterus recovery (Fig. 9). These findings provide novel evidence supporting the combined use of MB-exos and METTL3 inhibitors to regulate ESCs survival in endometrial repair following injury.

## Supplementary Information

Additional file 1. Fig. S1. miR-340/BMSCs identification. (A) Morphological characteristics of miR-340/BMSCs determined by optical microscopy. Scale bars: 20 μm. (B-I) Flow cytometry determination of surface markers in miR-340/BMSCs. (B-D) Isotype controls for FITC, PE, and FITC. miR-340/BMSCs showed negative staining for CD45 (E) and CD34 (F) but positive staining for CD29 (G), CD44 (H), and CD90 (I). Fig. S2. Detection of exosome markers by western blotting assay. (A) Calnexin, CD9, CD81, TSG101, CD63, and Hsp70 expression levels were assessed. Fig. S3. Procedure for mechanical damage of the endometrium and statistical analysis. (A) Schematic representation of MB-exos or B-exos treatment following mechanical damage to the endometrium. (B) Statistical analysis of endometrial thickness based on histological sections of the uterus (n = 6/group) [**P < 0.01, ****P < 0.0001]. (C) Statistical analysis of the fibrotic area percentage in the endometrium (**P < 0.01, ****P < 0.0001; n = 6/group). MD: mechanical damage. Fig. S4. Detection of cell senescence markers β-galactosidase and P21. (A) β-galactosidase expression levels were assessed using Cell Senescence β-Galactosidase Staining Kit. (B) P21 expression were assessed by immunohistochemistry. Fig. S5. Effects of the ferroptosis activator erastin on MB-exos in promoting the injured uterus recovery. (A) Representative images of uterus tissues of the Sham, PBS, MB-exos, and MB-exos+erastin groups (n = 6/group) stained with Masson’s trichrome stain. (B) Statistical analysis of the percentage of the endometrial fibrotic area in each group [***P < 0.001, ****P < 0.0001] (n = 6/group). (C) Statistical analysis of endometrial thickness based on histological sections of the uterus in each group (n = 6/group) [***P < 0.001, ****P < 0.0001]. (D-F) b-FGF, VEGF and IGF-1 levels in uterine tissue extracts from each group [*P < 0.05, **P < 0.01, ***P < 0.001, ****P < 0.0001] (n = 6/group). Fig. S6. B-exos or MB-exos impairs the inhibition of cell viability or lipid ROS production induced by RSL3 or erastin. (A) Primary endometrial stromal cell morphology was observed by optical microscopy. (B) Cell viability was determined by the Cell Counting Kit-8 for each group (control, erastin, B-exos+erastin, and MB-exos+erastin) [*P < 0.05, ****P < 0.0001] (n = 3/group). (C) Cell viability was quantified with the Cell Counting Kit-8 in each group (control, RSL3, B-exos+RSL3, and MB-exos+RSL3) (**P < 0.01, ****P < 0.0001; n = 3/group). Lipid ROS levels were estimated by C11-BODIPY (D) and analyzed (E) in each group (control, erastin, B-exos+erastin, and MB-exos+erastin) (*P < 0.05, ***P < 0.001, ****P < 0.0001; n = 3/group). Lipid ROS levels were detected using C11-BODIPY (F) and analyzed (G) in each group (control, RSL3, B-exos+RSL3, and MB-exos+RSL3) [**P < 0.01, ***P < 0.001, ****P < 0.0001] (n = 3/group). Fig. S7. METTL3 regulates RSL3-induced ferroptosis of ESCs. Knockdown of METTL3 suppressed RSL3-induced ferroptotic cell death (A-G). METTL3 shRNA was stably transfected into ESCs followed 24-h RSL3 (2.5 μM) treatment. (A) Fe2+ accumulation was quantified by an iron detection assay [*P < 0.05, ***P < 0.001, n.s.: not significant] (n = 3/group). (B) The lipid formation was measured by MDA assay [n.s.: not significant, **P < 0.01, ****P < 0.0001] (n = 3/group). GSH (C) and GSSG (D) levels were quantified by relative assay kits (n = 3/group) [n.s., not significant, *P < 0.05, **P < 0.01, ****P < 0.0001]. (E, F) Flow cytometry with C11-BODIPY was conducted for estimating the lipid ROS level (n = 3/group) [**P < 0.01, ***P < 0.001, n.s.: not significant]. (G) Cell viability was assessed with the Cell Counting Kit-8 [*P < 0.05, **P < 0.01] (n = 3/group). METTL3 overexpression enhanced RSL3-induced ferroptotic cell death (H-N). METTL3 plasmid was stably transfected into ESCs followed by 24-h RSL3 (2.5 μM) treatment. (H) Fe2+ accumulation was estimated by an iron detection assay [n.s.: not significant, **P < 0.01, ***P < 0.001] (n = 3/group). (I) Lipid formation was evaluated with MDA assay (n = 3/group) [*P < 0.05, **P < 0.01, n.s.: not significant]. GSH (J) and GSSG (K) levels were quantified by relative assay kits [*P < 0.05, **P < 0.01, ***P < 0.001, n.s.: not significant] (n = 3/group). (L-M) Flow cytometry with C11-BODIPY was carried out for estimating the lipid ROS level (n = 3/group) [**P < 0.01, n.s.: not significant]. (N) Cell Counting Kit-8 kit was utilized for estimating cell viability [**P < 0.01] (n = 3/group). Fig. S8. Detection of ROS level and mitochondrial morphology in ESCs transfected with HMOX1 shRNA and METTL3 shRNA followed by erastin treatment. (A) Flow cytometry with C11-BODIPY was conducted for quantifying the lipid ROS level [**P < 0.01, ***P < 0.001] (n = 3/group). (B) Mitochondrial morphology observed through transmission electron microscopy. Scale bars: 1 μm. Fig. S9. m6A binding site of HMOX1 mRNA.

Additional file 2.

Additional file 3.

## Data Availability

The RNA-seq dataset supporting the conclusions of this article is available in the Sequence Read Archive (SRA) repository under study accession PRJNA1129286.
