# Correction: Mir-340-3p-modified bone marrow mesenchymal stem cell-derived exosomes inhibit ferroptosis through METTL3-mediated m6A modification of HMOX1 to promote recovery of injured rat uterus

**DOI:** 10.1186/s13287-024-03985-w

**Published:** 2024-10-10

**Authors:** Bang Xiao, Yiqing Zhu, Meng Liu, Meiting Chen, Chao Huang, Dabing Xu, Fang Wang, Shuhan Sun, Jinfeng Huang, Ningxia Sun, Fu Yang

**Affiliations:** 1 Department of Medical Genetics, Naval Medical University, Shanghai, 200433 China; 2 Department of Anatomy, Institute of Biomedical Engineering, Naval Medical University, Shanghai, 200433 China; 3 The Center of Reproductive Medicine, Second Affiliated Hospital of Naval Medical University, Shanghai, 200003 China

**Correction to: Stem Cell Research & Therapy (2024) 15:224**

10.1186/s13287-024-03846-6

The original article erroneously presents an overlapping artefact in Fig. 2H; the corrected figure can be viewed ahead in this Correction article.

**Fig. 2:**
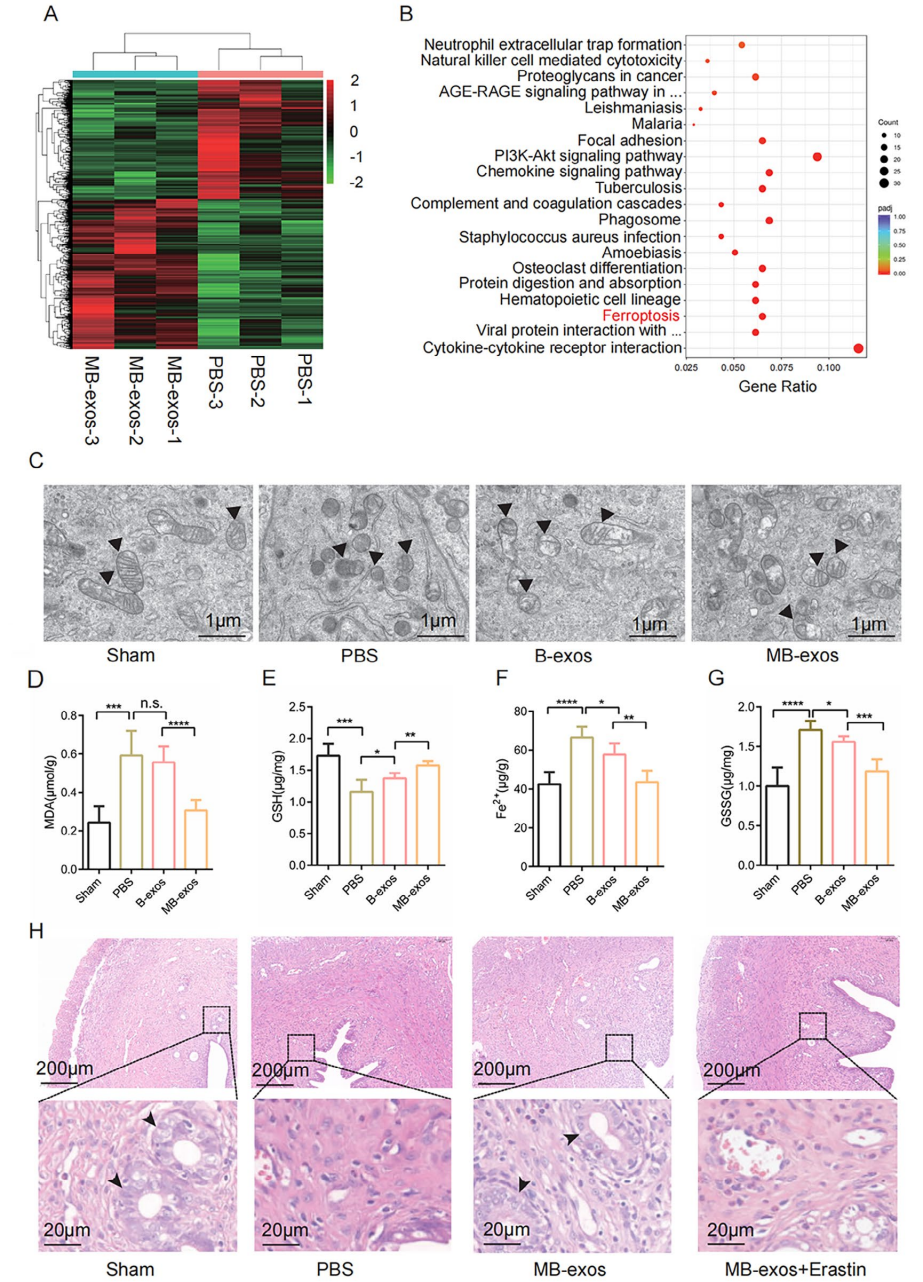
MB-exos promote recovery of the injured uterus through the inhibition of ferroptosis. **A** Heatmap displaying the DEGs between the PBS and MB-exos groups (n = 3). **B** DEGs were evaluated with KEGG analysis, and 20 significantly enriched pathways are presented. **C** Transmission electron microscopy images showing mitochondrial morphology in the endometrium of different groups (Sham, PBS, B-exos, and MB-exos) (n = 3/group). **D**–**G** Assay results for MDA production, GSH depletion, iron accumulation, and GSSG levels in each group (Sham, PBS, B-exos, and MB-exos) [n.s.: not significant, *P < 0.05, **P < 0.01, ***P < 0.001, ****P < 0.0001] (n = 6/group). **H** Representative images of hematoxylin and eosin (HE)-stained uterus tissues from the Sham, PBS, MB-exos, and MB-exos + erastin groups (n = 6/group)

