# Supplementary figures and images for: miR-340-3p-modified bone marrow mesenchymal stem cell-derived exosomes inhibit ferroptosis through METTL3-mediated m6A modification of HMOX1 to promote recovery of injured rat uterus
Source: Stem Cell Res Ther. 2024 Jul 29;15:224. doi: 10.1186/s13287-024-03846-6 (PMC11287883; doi:10.1186/s13287-024-03846-6)

A

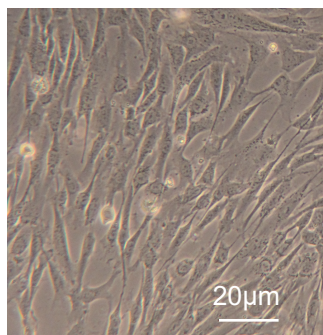

B

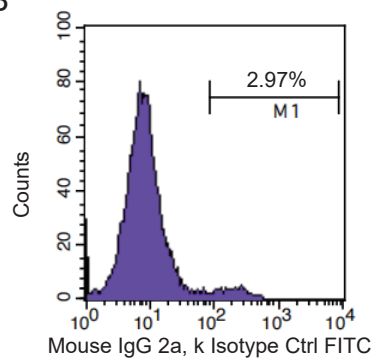

C

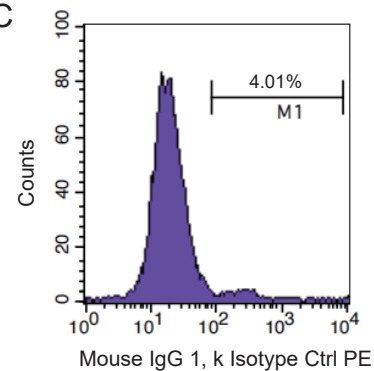

D

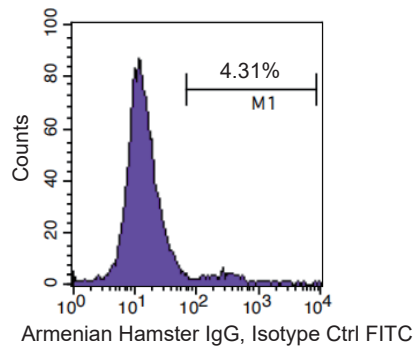

E

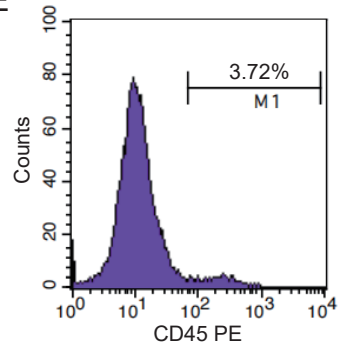

F

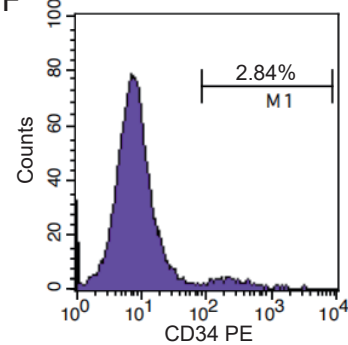

G

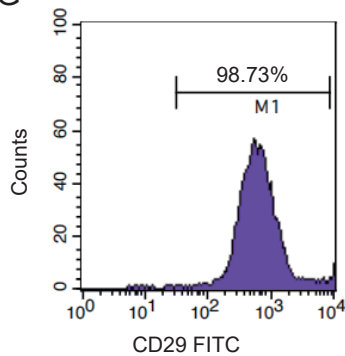

H

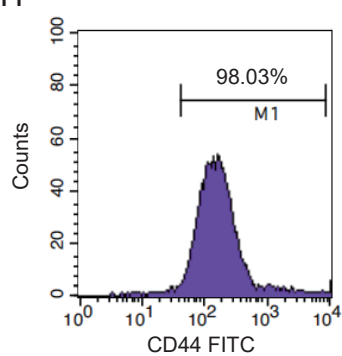

I

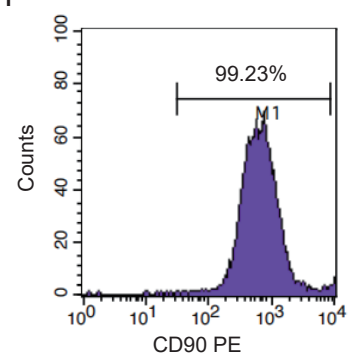

Supplement: Supplementary file 1 — Additional file 1. Fig. S1. miR-340/BMSCs identification. (A) Morphological characteristics of miR-340/BMSCs determined by optical microscopy. Scale bars: 20 μm. (B-I) Flow cytometry determination of surface markers in miR-340/BMSCs. (B-D) Isotype controls for FITC, PE, and FITC. miR-340/BMSCs showed negative staining for CD45 (E) and CD34 (F) but positive staining for CD29 (G), CD44 (H), and CD90 (I). Fig. S2. Detection of exosome markers by western blotting assay. (A) Calnexin, CD9, CD81, TSG101, CD63, and Hsp70 expression levels were assessed. Fig. S3. Procedure for mechanical damage of the endometrium and statistical analysis. (A) Schematic representation of MB-exos or B-exos treatment following mechanical damage to the endometrium. (B) Statistical analysis of endometrial thickness based on histological sections of the uterus (n = 6/group) [**P < 0.01, ****P < 0.0001]. (C) Statistical analysis of the fibrotic area percentage in the endometrium (**P < 0.01, ****P < 0.0001; n = 6/group). MD: mechanical damage. Fig. S4. Detection of cell senescence markers β-galactosidase and P21. (A) β-galactosidase expression levels were assessed using Cell Senescence β-Galactosidase Staining Kit. (B) P21 expression were assessed by immunohistochemistry. Fig. S5. Effects of the ferroptosis activator erastin on MB-exos in promoting the injured uterus recovery. (A) Representative images of uterus tissues of the Sham, PBS, MB-exos, and MB-exos+erastin groups (n = 6/group) stained with Masson’s trichrome stain. (B) Statistical analysis of the percentage of the endometrial fibrotic area in each group [***P < 0.001, ****P < 0.0001] (n = 6/group). (C) Statistical analysis of endometrial thickness based on histological sections of the uterus in each group (n = 6/group) [***P < 0.001, ****P < 0.0001]. (D-F) b-FGF, VEGF and IGF-1 levels in uterine tissue extracts from each group [*P < 0.05, **P < 0.01, ***P < 0.001, ****P < 0.0001] (n = 6/group). Fig. S6. B-exos or MB-exos impairs the inhi [file 13287_2024_3846_MOESM1_ESM.zip › 13287/figure S1.pdf]

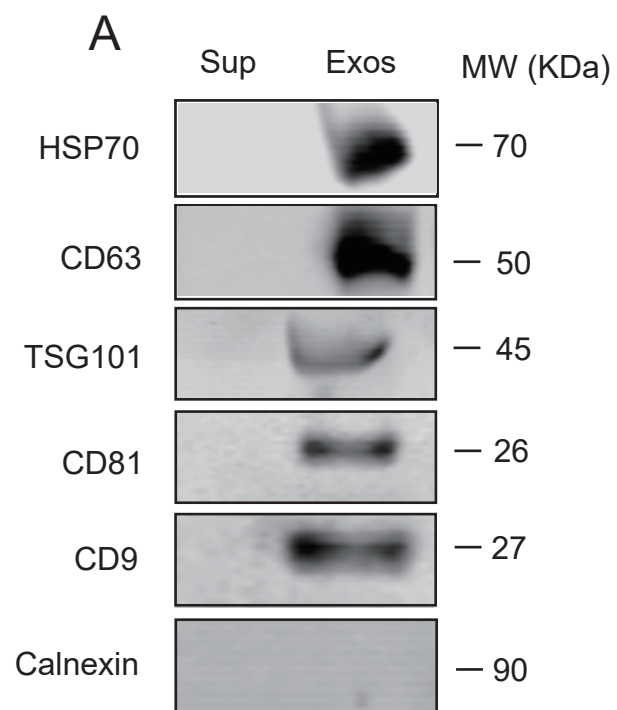

Supplement: Supplementary file 1 — Additional file 1. Fig. S1. miR-340/BMSCs identification. (A) Morphological characteristics of miR-340/BMSCs determined by optical microscopy. Scale bars: 20 μm. (B-I) Flow cytometry determination of surface markers in miR-340/BMSCs. (B-D) Isotype controls for FITC, PE, and FITC. miR-340/BMSCs showed negative staining for CD45 (E) and CD34 (F) but positive staining for CD29 (G), CD44 (H), and CD90 (I). Fig. S2. Detection of exosome markers by western blotting assay. (A) Calnexin, CD9, CD81, TSG101, CD63, and Hsp70 expression levels were assessed. Fig. S3. Procedure for mechanical damage of the endometrium and statistical analysis. (A) Schematic representation of MB-exos or B-exos treatment following mechanical damage to the endometrium. (B) Statistical analysis of endometrial thickness based on histological sections of the uterus (n = 6/group) [**P < 0.01, ****P < 0.0001]. (C) Statistical analysis of the fibrotic area percentage in the endometrium (**P < 0.01, ****P < 0.0001; n = 6/group). MD: mechanical damage. Fig. S4. Detection of cell senescence markers β-galactosidase and P21. (A) β-galactosidase expression levels were assessed using Cell Senescence β-Galactosidase Staining Kit. (B) P21 expression were assessed by immunohistochemistry. Fig. S5. Effects of the ferroptosis activator erastin on MB-exos in promoting the injured uterus recovery. (A) Representative images of uterus tissues of the Sham, PBS, MB-exos, and MB-exos+erastin groups (n = 6/group) stained with Masson’s trichrome stain. (B) Statistical analysis of the percentage of the endometrial fibrotic area in each group [***P < 0.001, ****P < 0.0001] (n = 6/group). (C) Statistical analysis of endometrial thickness based on histological sections of the uterus in each group (n = 6/group) [***P < 0.001, ****P < 0.0001]. (D-F) b-FGF, VEGF and IGF-1 levels in uterine tissue extracts from each group [*P < 0.05, **P < 0.01, ***P < 0.001, ****P < 0.0001] (n = 6/group). Fig. S6. B-exos or MB-exos impairs the inhi [file 13287_2024_3846_MOESM1_ESM.zip › 13287/figure S2.pdf]

A

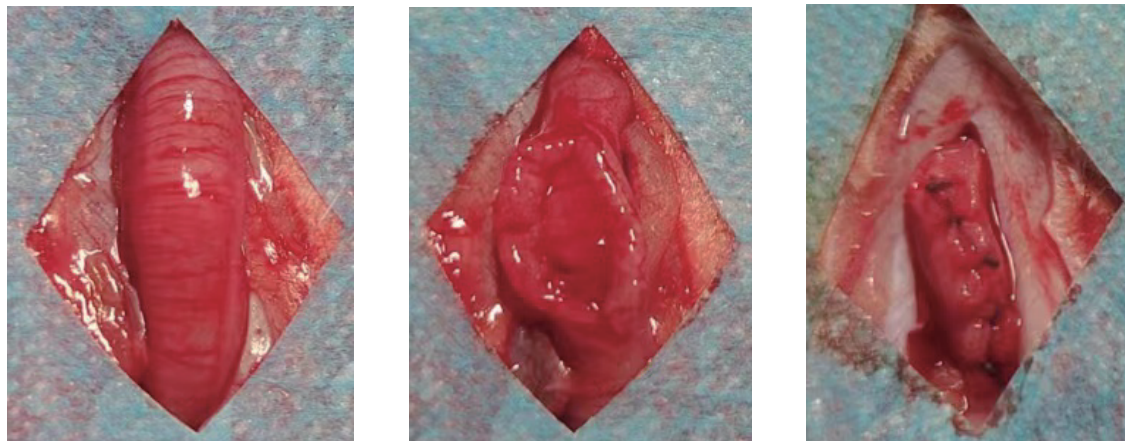

MD

B

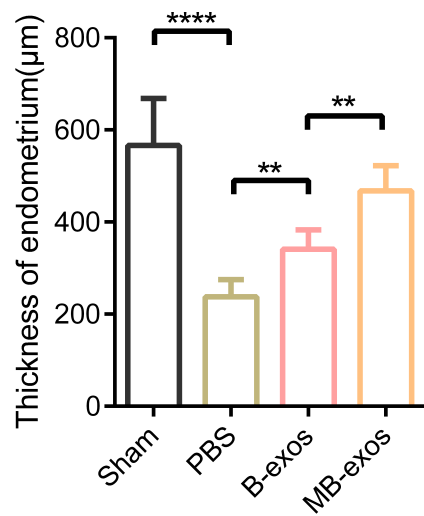

C

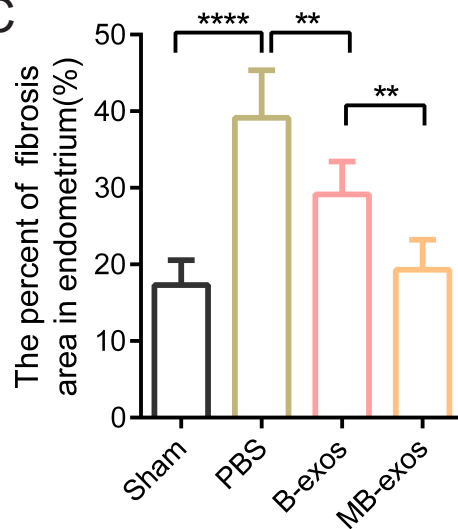

Supplement: Supplementary file 1 — Additional file 1. Fig. S1. miR-340/BMSCs identification. (A) Morphological characteristics of miR-340/BMSCs determined by optical microscopy. Scale bars: 20 μm. (B-I) Flow cytometry determination of surface markers in miR-340/BMSCs. (B-D) Isotype controls for FITC, PE, and FITC. miR-340/BMSCs showed negative staining for CD45 (E) and CD34 (F) but positive staining for CD29 (G), CD44 (H), and CD90 (I). Fig. S2. Detection of exosome markers by western blotting assay. (A) Calnexin, CD9, CD81, TSG101, CD63, and Hsp70 expression levels were assessed. Fig. S3. Procedure for mechanical damage of the endometrium and statistical analysis. (A) Schematic representation of MB-exos or B-exos treatment following mechanical damage to the endometrium. (B) Statistical analysis of endometrial thickness based on histological sections of the uterus (n = 6/group) [**P < 0.01, ****P < 0.0001]. (C) Statistical analysis of the fibrotic area percentage in the endometrium (**P < 0.01, ****P < 0.0001; n = 6/group). MD: mechanical damage. Fig. S4. Detection of cell senescence markers β-galactosidase and P21. (A) β-galactosidase expression levels were assessed using Cell Senescence β-Galactosidase Staining Kit. (B) P21 expression were assessed by immunohistochemistry. Fig. S5. Effects of the ferroptosis activator erastin on MB-exos in promoting the injured uterus recovery. (A) Representative images of uterus tissues of the Sham, PBS, MB-exos, and MB-exos+erastin groups (n = 6/group) stained with Masson’s trichrome stain. (B) Statistical analysis of the percentage of the endometrial fibrotic area in each group [***P < 0.001, ****P < 0.0001] (n = 6/group). (C) Statistical analysis of endometrial thickness based on histological sections of the uterus in each group (n = 6/group) [***P < 0.001, ****P < 0.0001]. (D-F) b-FGF, VEGF and IGF-1 levels in uterine tissue extracts from each group [*P < 0.05, **P < 0.01, ***P < 0.001, ****P < 0.0001] (n = 6/group). Fig. S6. B-exos or MB-exos impairs the inhi [file 13287_2024_3846_MOESM1_ESM.zip › 13287/figure S3.pdf]

**A** **$\beta$ -Galactosidase Staining****Sham**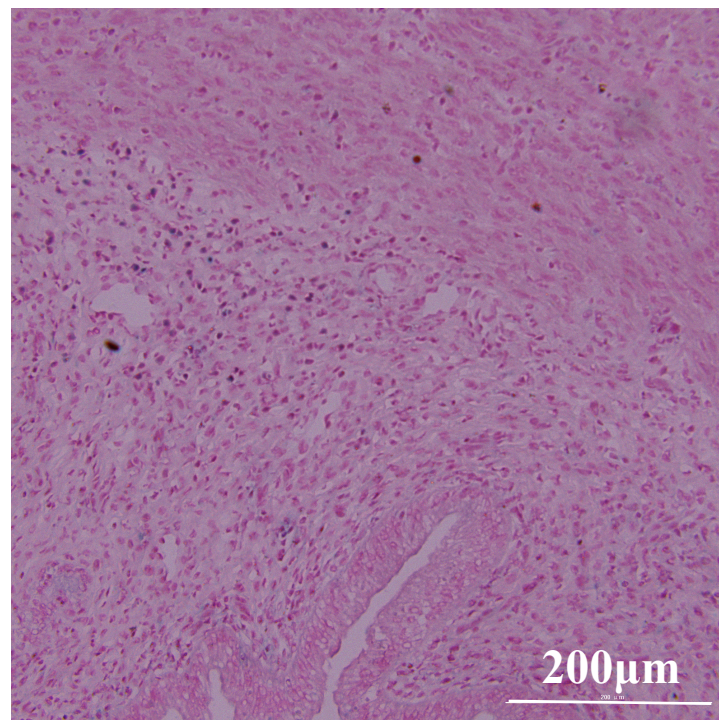**PBS**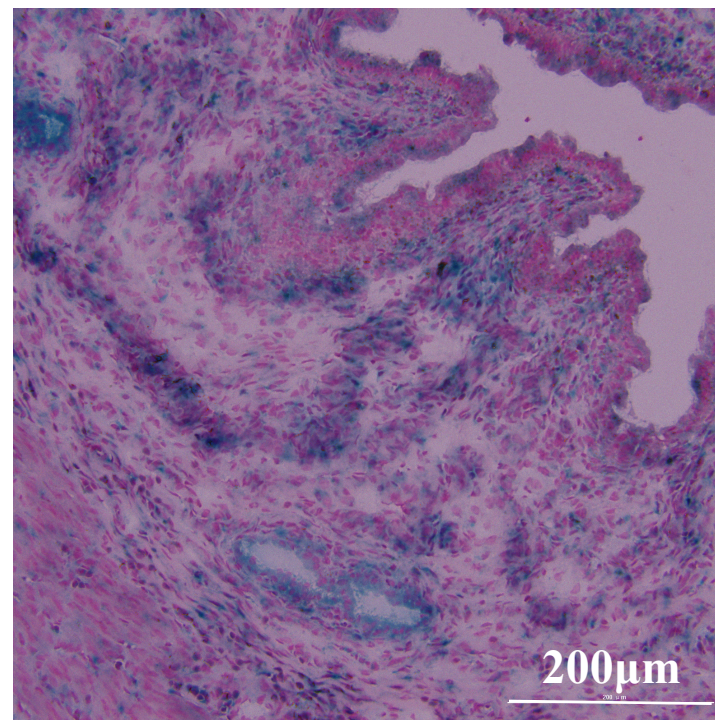**B-exos**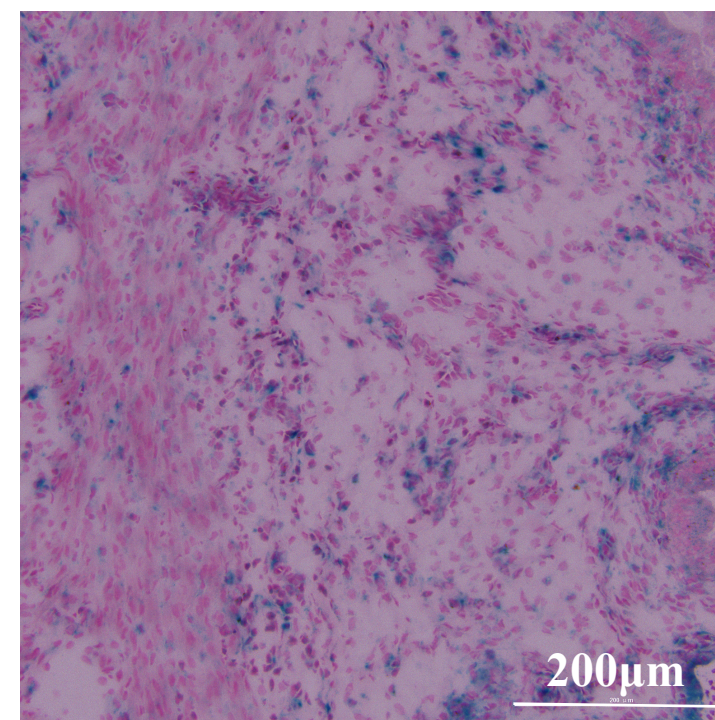**MB-exos**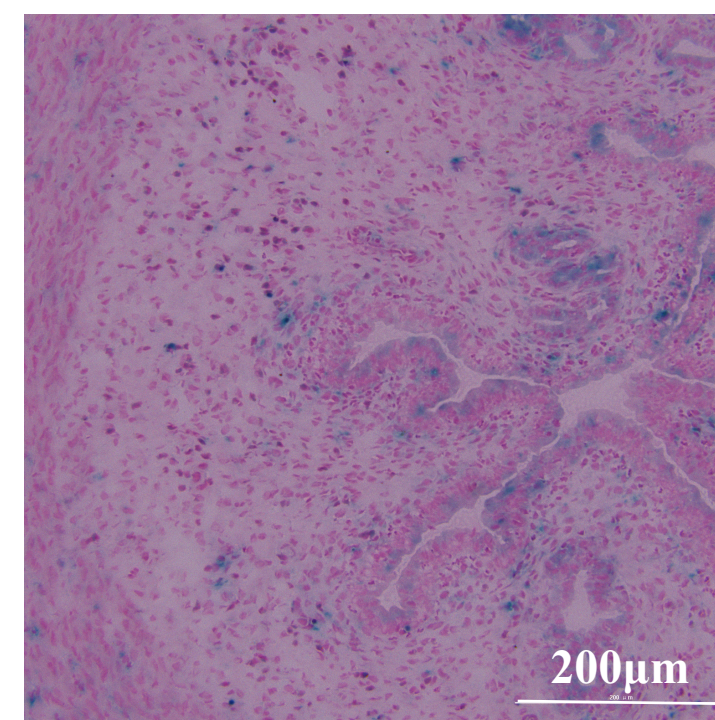**B****IHC for P21****Sham**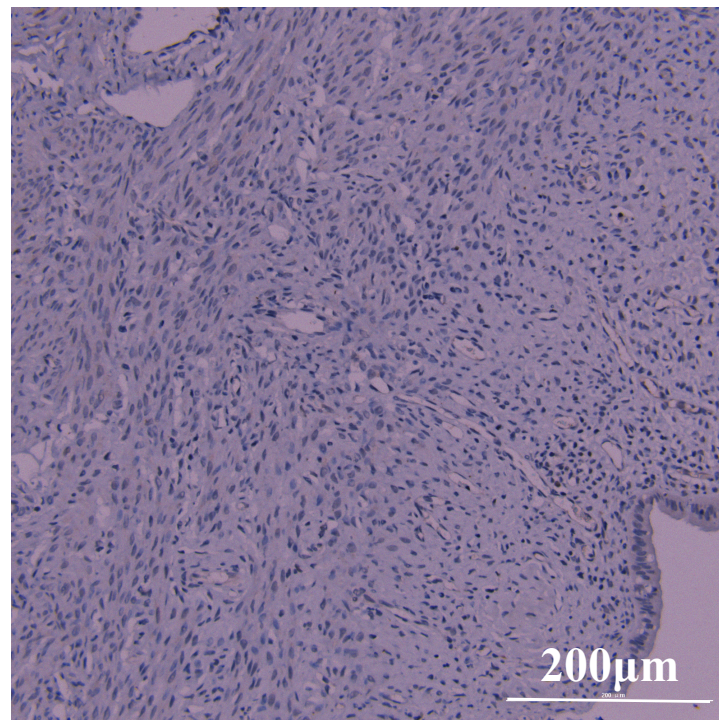**PBS**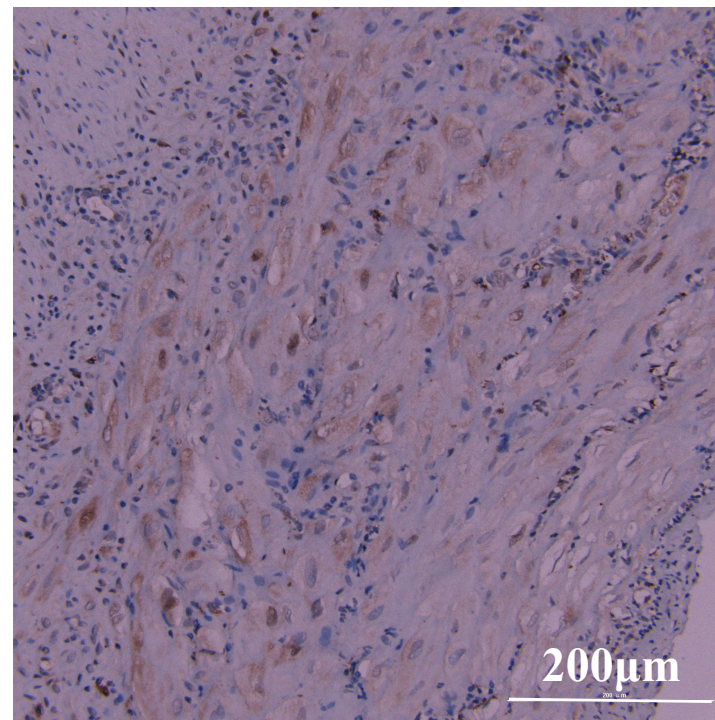**B-exos**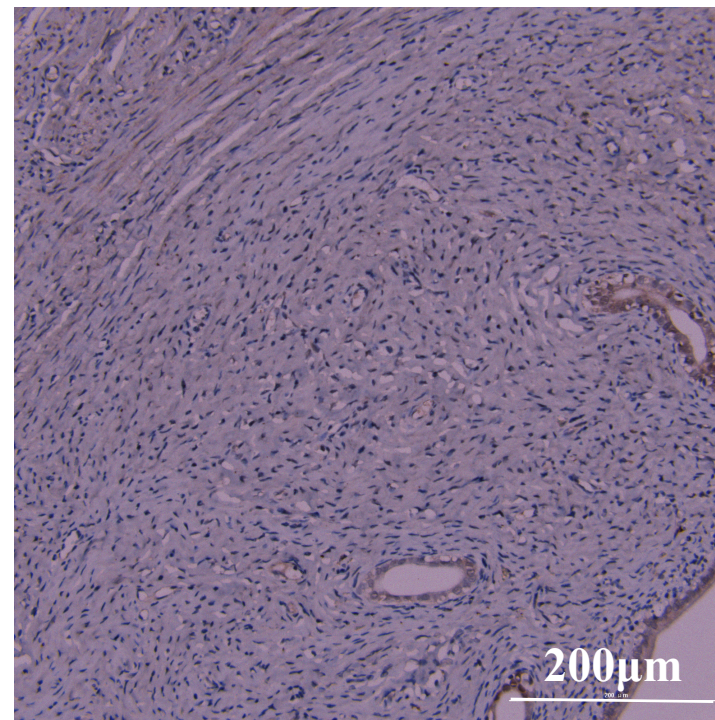**MB-exos**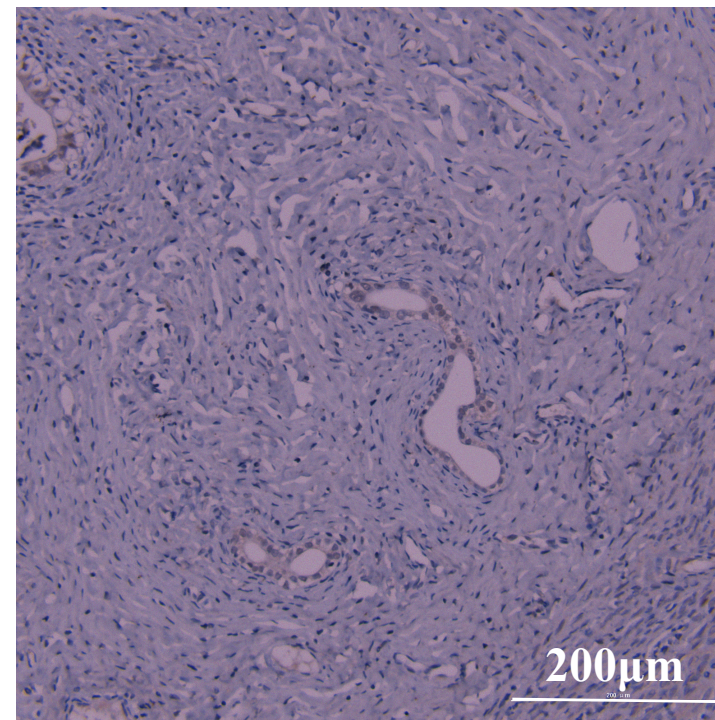

Supplement: Supplementary file 1 — Additional file 1. Fig. S1. miR-340/BMSCs identification. (A) Morphological characteristics of miR-340/BMSCs determined by optical microscopy. Scale bars: 20 μm. (B-I) Flow cytometry determination of surface markers in miR-340/BMSCs. (B-D) Isotype controls for FITC, PE, and FITC. miR-340/BMSCs showed negative staining for CD45 (E) and CD34 (F) but positive staining for CD29 (G), CD44 (H), and CD90 (I). Fig. S2. Detection of exosome markers by western blotting assay. (A) Calnexin, CD9, CD81, TSG101, CD63, and Hsp70 expression levels were assessed. Fig. S3. Procedure for mechanical damage of the endometrium and statistical analysis. (A) Schematic representation of MB-exos or B-exos treatment following mechanical damage to the endometrium. (B) Statistical analysis of endometrial thickness based on histological sections of the uterus (n = 6/group) [**P < 0.01, ****P < 0.0001]. (C) Statistical analysis of the fibrotic area percentage in the endometrium (**P < 0.01, ****P < 0.0001; n = 6/group). MD: mechanical damage. Fig. S4. Detection of cell senescence markers β-galactosidase and P21. (A) β-galactosidase expression levels were assessed using Cell Senescence β-Galactosidase Staining Kit. (B) P21 expression were assessed by immunohistochemistry. Fig. S5. Effects of the ferroptosis activator erastin on MB-exos in promoting the injured uterus recovery. (A) Representative images of uterus tissues of the Sham, PBS, MB-exos, and MB-exos+erastin groups (n = 6/group) stained with Masson’s trichrome stain. (B) Statistical analysis of the percentage of the endometrial fibrotic area in each group [***P < 0.001, ****P < 0.0001] (n = 6/group). (C) Statistical analysis of endometrial thickness based on histological sections of the uterus in each group (n = 6/group) [***P < 0.001, ****P < 0.0001]. (D-F) b-FGF, VEGF and IGF-1 levels in uterine tissue extracts from each group [*P < 0.05, **P < 0.01, ***P < 0.001, ****P < 0.0001] (n = 6/group). Fig. S6. B-exos or MB-exos impairs the inhi [file 13287_2024_3846_MOESM1_ESM.zip › 13287/figure S4.pdf]

A

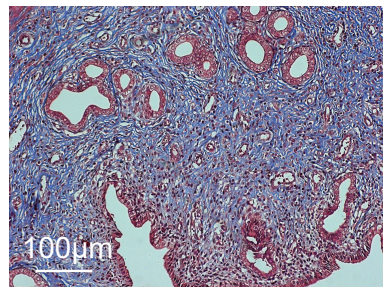

Sham

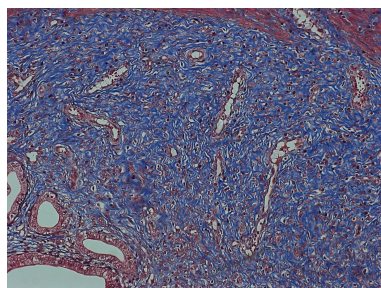

PBS

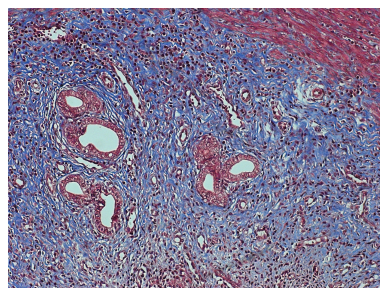

MB-exos

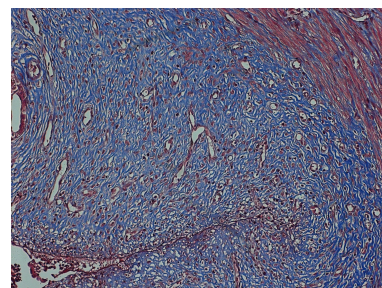

MB-exos+Erastin

B

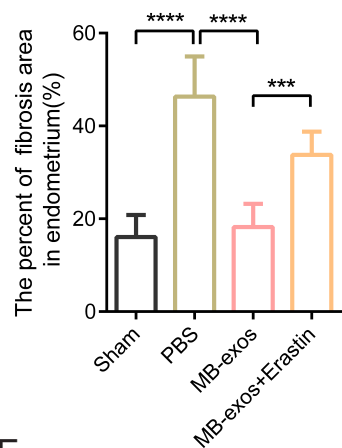

C

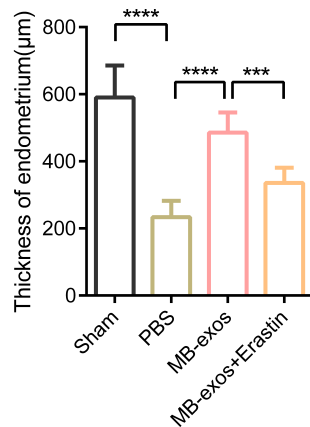

D

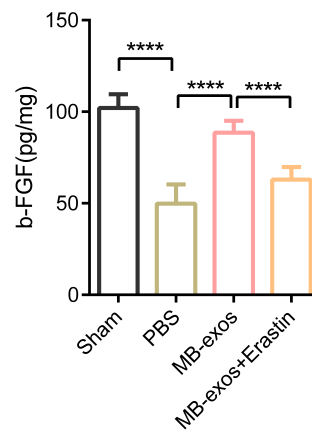

E

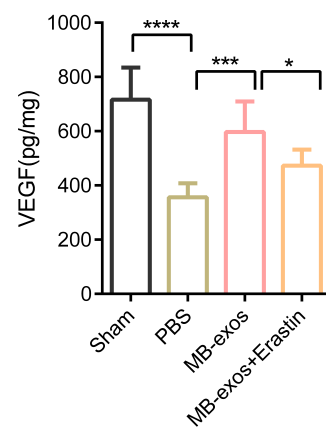

F

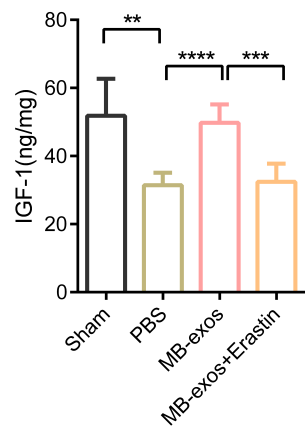

Supplement: Supplementary file 1 — Additional file 1. Fig. S1. miR-340/BMSCs identification. (A) Morphological characteristics of miR-340/BMSCs determined by optical microscopy. Scale bars: 20 μm. (B-I) Flow cytometry determination of surface markers in miR-340/BMSCs. (B-D) Isotype controls for FITC, PE, and FITC. miR-340/BMSCs showed negative staining for CD45 (E) and CD34 (F) but positive staining for CD29 (G), CD44 (H), and CD90 (I). Fig. S2. Detection of exosome markers by western blotting assay. (A) Calnexin, CD9, CD81, TSG101, CD63, and Hsp70 expression levels were assessed. Fig. S3. Procedure for mechanical damage of the endometrium and statistical analysis. (A) Schematic representation of MB-exos or B-exos treatment following mechanical damage to the endometrium. (B) Statistical analysis of endometrial thickness based on histological sections of the uterus (n = 6/group) [**P < 0.01, ****P < 0.0001]. (C) Statistical analysis of the fibrotic area percentage in the endometrium (**P < 0.01, ****P < 0.0001; n = 6/group). MD: mechanical damage. Fig. S4. Detection of cell senescence markers β-galactosidase and P21. (A) β-galactosidase expression levels were assessed using Cell Senescence β-Galactosidase Staining Kit. (B) P21 expression were assessed by immunohistochemistry. Fig. S5. Effects of the ferroptosis activator erastin on MB-exos in promoting the injured uterus recovery. (A) Representative images of uterus tissues of the Sham, PBS, MB-exos, and MB-exos+erastin groups (n = 6/group) stained with Masson’s trichrome stain. (B) Statistical analysis of the percentage of the endometrial fibrotic area in each group [***P < 0.001, ****P < 0.0001] (n = 6/group). (C) Statistical analysis of endometrial thickness based on histological sections of the uterus in each group (n = 6/group) [***P < 0.001, ****P < 0.0001]. (D-F) b-FGF, VEGF and IGF-1 levels in uterine tissue extracts from each group [*P < 0.05, **P < 0.01, ***P < 0.001, ****P < 0.0001] (n = 6/group). Fig. S6. B-exos or MB-exos impairs the inhi [file 13287_2024_3846_MOESM1_ESM.zip › 13287/figure S5.pdf]

A

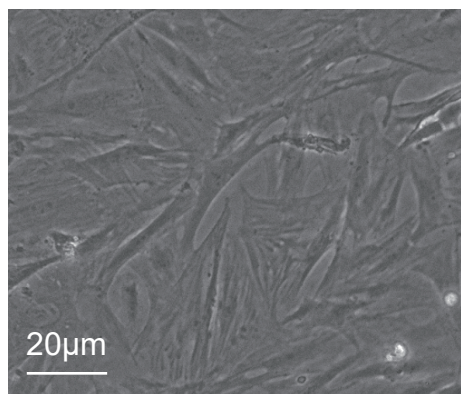

B

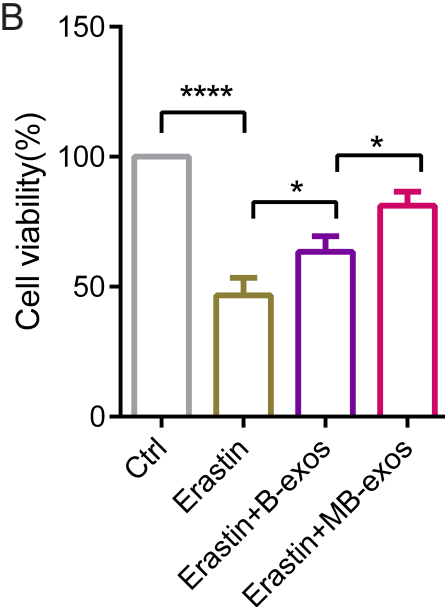

C

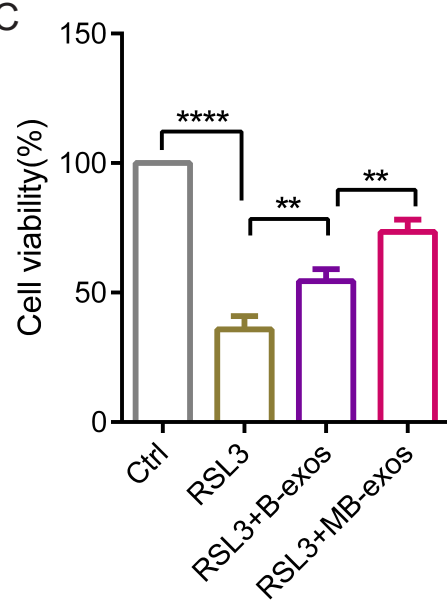

D

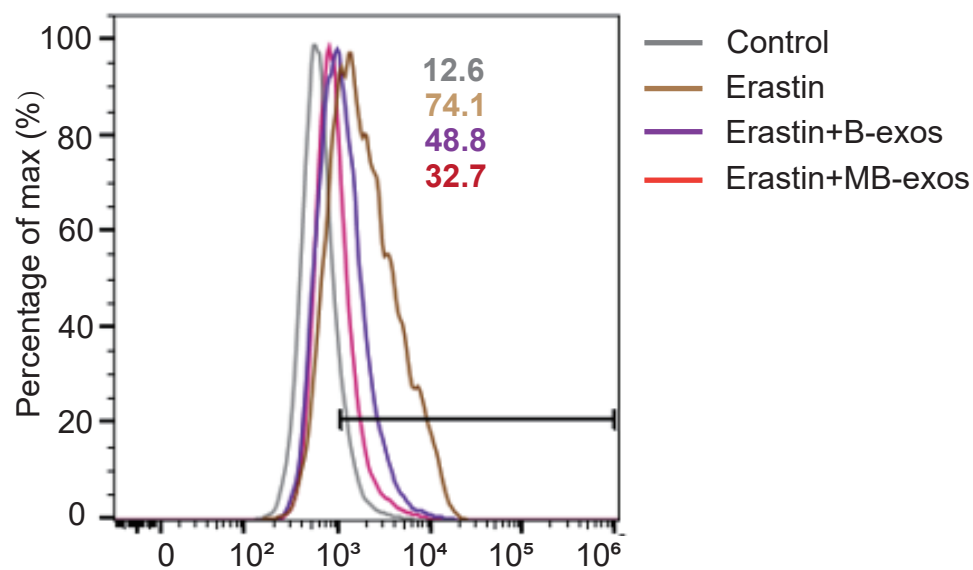

E

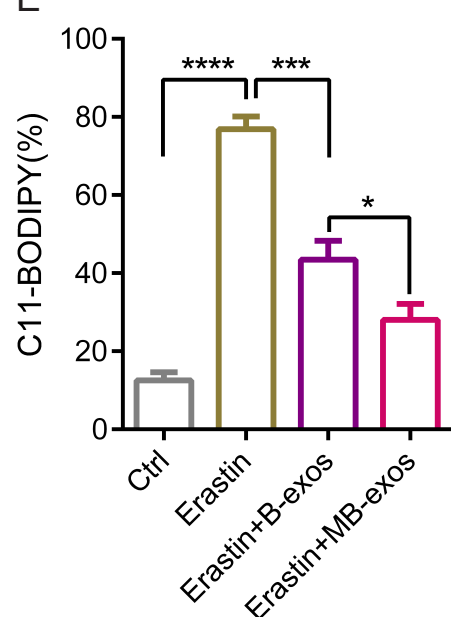

F

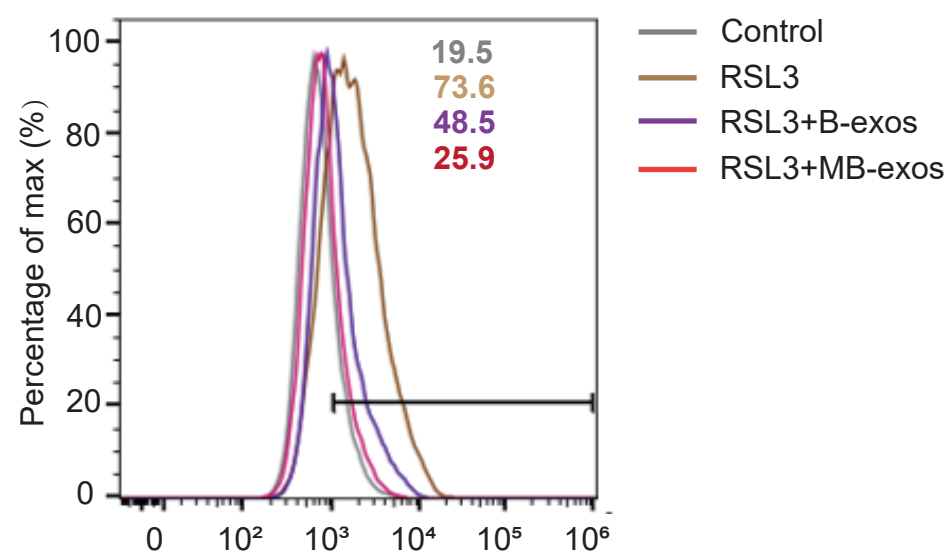

G

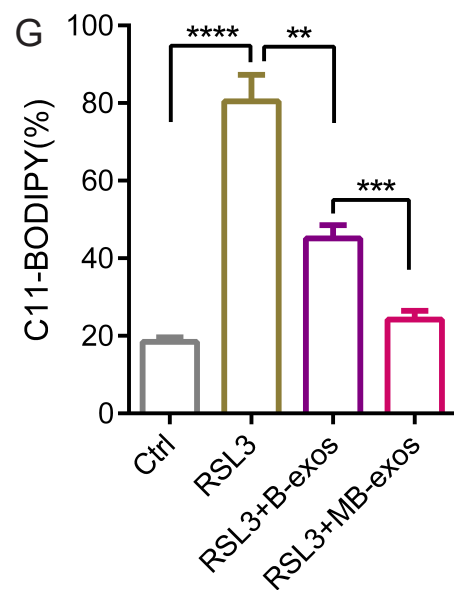

Supplement: Supplementary file 1 — Additional file 1. Fig. S1. miR-340/BMSCs identification. (A) Morphological characteristics of miR-340/BMSCs determined by optical microscopy. Scale bars: 20 μm. (B-I) Flow cytometry determination of surface markers in miR-340/BMSCs. (B-D) Isotype controls for FITC, PE, and FITC. miR-340/BMSCs showed negative staining for CD45 (E) and CD34 (F) but positive staining for CD29 (G), CD44 (H), and CD90 (I). Fig. S2. Detection of exosome markers by western blotting assay. (A) Calnexin, CD9, CD81, TSG101, CD63, and Hsp70 expression levels were assessed. Fig. S3. Procedure for mechanical damage of the endometrium and statistical analysis. (A) Schematic representation of MB-exos or B-exos treatment following mechanical damage to the endometrium. (B) Statistical analysis of endometrial thickness based on histological sections of the uterus (n = 6/group) [**P < 0.01, ****P < 0.0001]. (C) Statistical analysis of the fibrotic area percentage in the endometrium (**P < 0.01, ****P < 0.0001; n = 6/group). MD: mechanical damage. Fig. S4. Detection of cell senescence markers β-galactosidase and P21. (A) β-galactosidase expression levels were assessed using Cell Senescence β-Galactosidase Staining Kit. (B) P21 expression were assessed by immunohistochemistry. Fig. S5. Effects of the ferroptosis activator erastin on MB-exos in promoting the injured uterus recovery. (A) Representative images of uterus tissues of the Sham, PBS, MB-exos, and MB-exos+erastin groups (n = 6/group) stained with Masson’s trichrome stain. (B) Statistical analysis of the percentage of the endometrial fibrotic area in each group [***P < 0.001, ****P < 0.0001] (n = 6/group). (C) Statistical analysis of endometrial thickness based on histological sections of the uterus in each group (n = 6/group) [***P < 0.001, ****P < 0.0001]. (D-F) b-FGF, VEGF and IGF-1 levels in uterine tissue extracts from each group [*P < 0.05, **P < 0.01, ***P < 0.001, ****P < 0.0001] (n = 6/group). Fig. S6. B-exos or MB-exos impairs the inhi [file 13287_2024_3846_MOESM1_ESM.zip › 13287/figure S6.pdf]

A

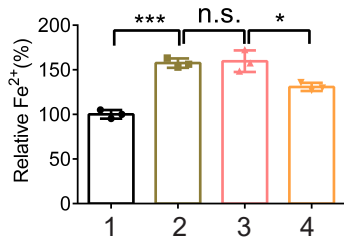

B

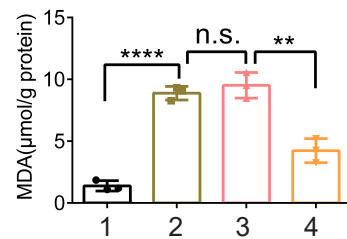

C

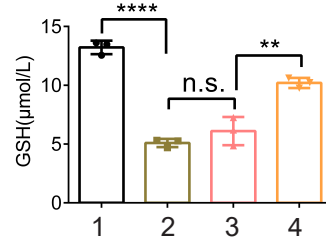

D

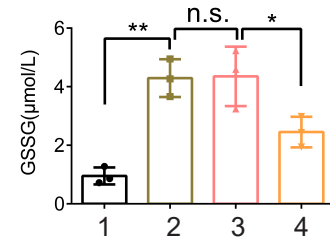

E

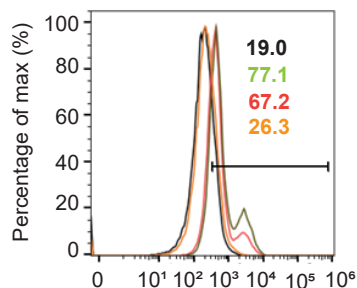

F

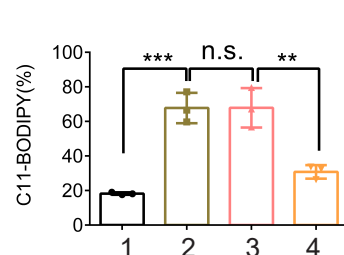

G

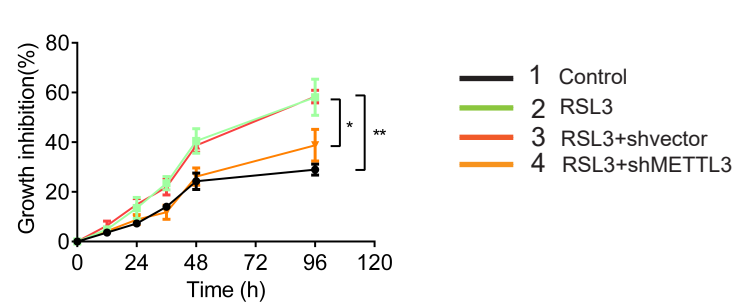

H

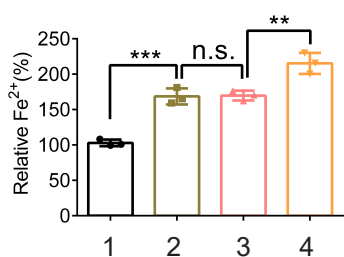

I

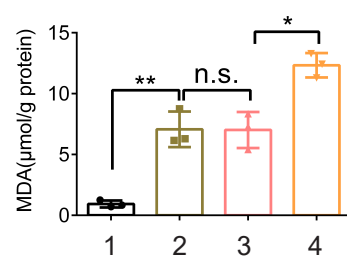

J

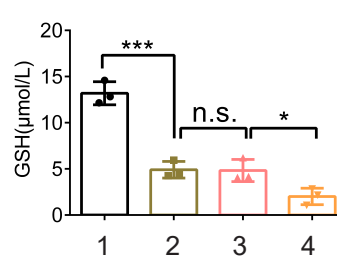

K

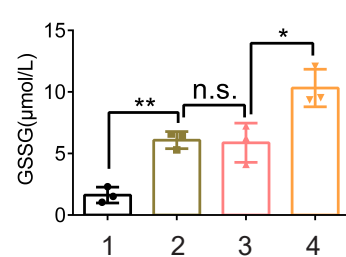

L

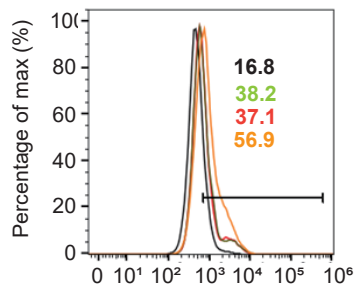

M

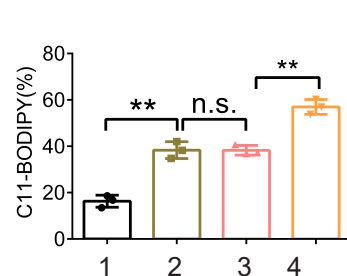

N

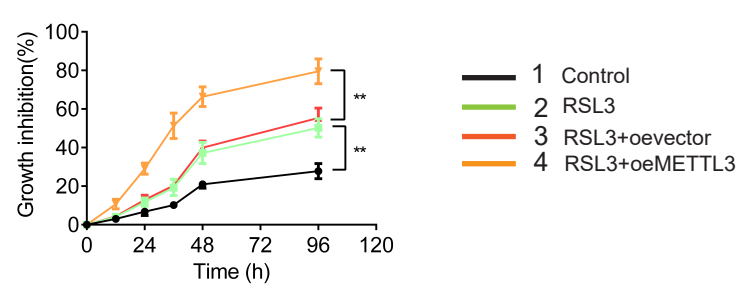

Supplement: Supplementary file 1 — Additional file 1. Fig. S1. miR-340/BMSCs identification. (A) Morphological characteristics of miR-340/BMSCs determined by optical microscopy. Scale bars: 20 μm. (B-I) Flow cytometry determination of surface markers in miR-340/BMSCs. (B-D) Isotype controls for FITC, PE, and FITC. miR-340/BMSCs showed negative staining for CD45 (E) and CD34 (F) but positive staining for CD29 (G), CD44 (H), and CD90 (I). Fig. S2. Detection of exosome markers by western blotting assay. (A) Calnexin, CD9, CD81, TSG101, CD63, and Hsp70 expression levels were assessed. Fig. S3. Procedure for mechanical damage of the endometrium and statistical analysis. (A) Schematic representation of MB-exos or B-exos treatment following mechanical damage to the endometrium. (B) Statistical analysis of endometrial thickness based on histological sections of the uterus (n = 6/group) [**P < 0.01, ****P < 0.0001]. (C) Statistical analysis of the fibrotic area percentage in the endometrium (**P < 0.01, ****P < 0.0001; n = 6/group). MD: mechanical damage. Fig. S4. Detection of cell senescence markers β-galactosidase and P21. (A) β-galactosidase expression levels were assessed using Cell Senescence β-Galactosidase Staining Kit. (B) P21 expression were assessed by immunohistochemistry. Fig. S5. Effects of the ferroptosis activator erastin on MB-exos in promoting the injured uterus recovery. (A) Representative images of uterus tissues of the Sham, PBS, MB-exos, and MB-exos+erastin groups (n = 6/group) stained with Masson’s trichrome stain. (B) Statistical analysis of the percentage of the endometrial fibrotic area in each group [***P < 0.001, ****P < 0.0001] (n = 6/group). (C) Statistical analysis of endometrial thickness based on histological sections of the uterus in each group (n = 6/group) [***P < 0.001, ****P < 0.0001]. (D-F) b-FGF, VEGF and IGF-1 levels in uterine tissue extracts from each group [*P < 0.05, **P < 0.01, ***P < 0.001, ****P < 0.0001] (n = 6/group). Fig. S6. B-exos or MB-exos impairs the inhi [file 13287_2024_3846_MOESM1_ESM.zip › 13287/figure S7.pdf]

A

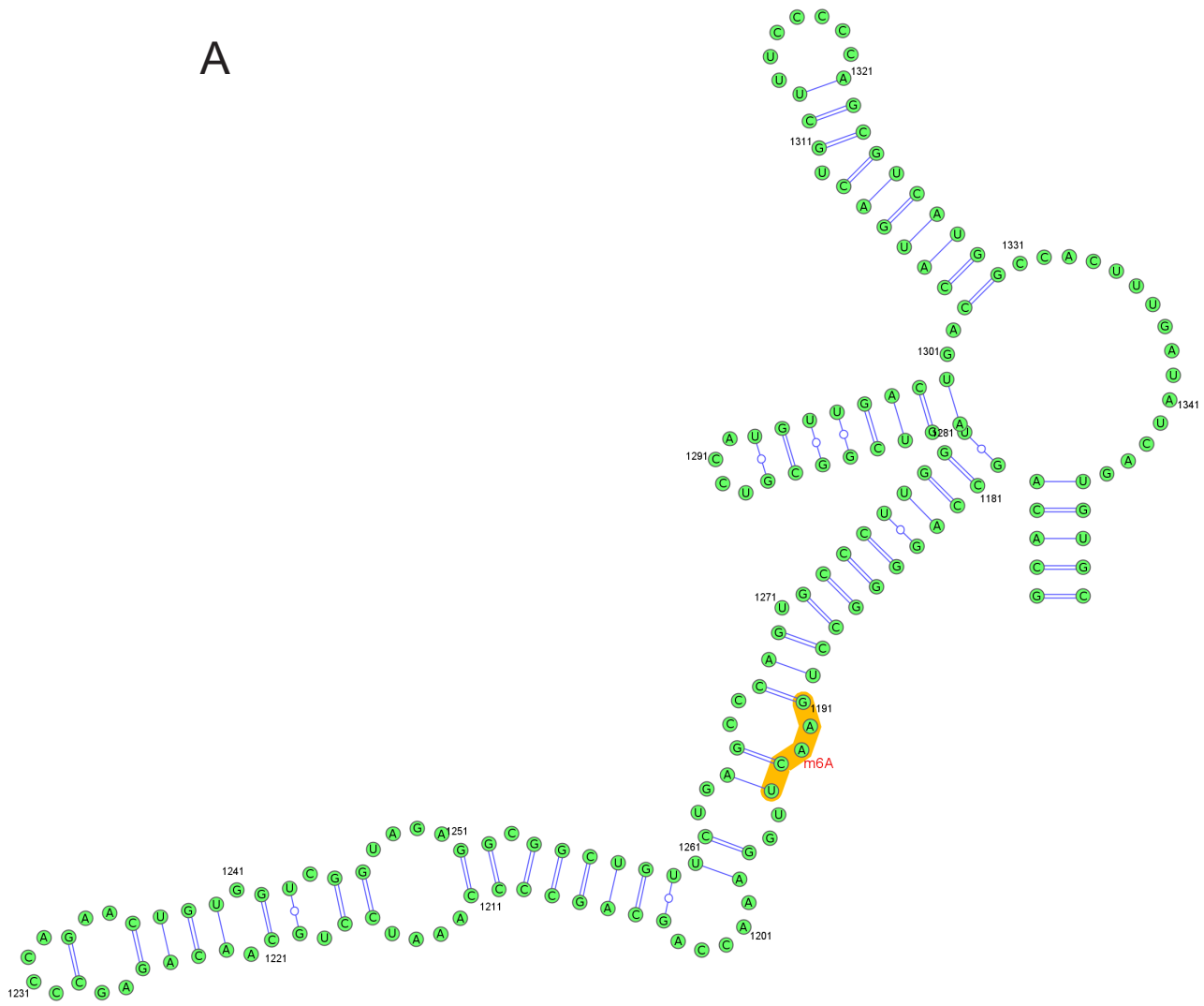

(HMOX1 A1193)

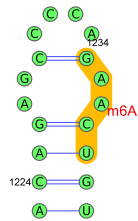

(HMOX1 A1236)

Supplement: Supplementary file 1 — Additional file 1. Fig. S1. miR-340/BMSCs identification. (A) Morphological characteristics of miR-340/BMSCs determined by optical microscopy. Scale bars: 20 μm. (B-I) Flow cytometry determination of surface markers in miR-340/BMSCs. (B-D) Isotype controls for FITC, PE, and FITC. miR-340/BMSCs showed negative staining for CD45 (E) and CD34 (F) but positive staining for CD29 (G), CD44 (H), and CD90 (I). Fig. S2. Detection of exosome markers by western blotting assay. (A) Calnexin, CD9, CD81, TSG101, CD63, and Hsp70 expression levels were assessed. Fig. S3. Procedure for mechanical damage of the endometrium and statistical analysis. (A) Schematic representation of MB-exos or B-exos treatment following mechanical damage to the endometrium. (B) Statistical analysis of endometrial thickness based on histological sections of the uterus (n = 6/group) [**P < 0.01, ****P < 0.0001]. (C) Statistical analysis of the fibrotic area percentage in the endometrium (**P < 0.01, ****P < 0.0001; n = 6/group). MD: mechanical damage. Fig. S4. Detection of cell senescence markers β-galactosidase and P21. (A) β-galactosidase expression levels were assessed using Cell Senescence β-Galactosidase Staining Kit. (B) P21 expression were assessed by immunohistochemistry. Fig. S5. Effects of the ferroptosis activator erastin on MB-exos in promoting the injured uterus recovery. (A) Representative images of uterus tissues of the Sham, PBS, MB-exos, and MB-exos+erastin groups (n = 6/group) stained with Masson’s trichrome stain. (B) Statistical analysis of the percentage of the endometrial fibrotic area in each group [***P < 0.001, ****P < 0.0001] (n = 6/group). (C) Statistical analysis of endometrial thickness based on histological sections of the uterus in each group (n = 6/group) [***P < 0.001, ****P < 0.0001]. (D-F) b-FGF, VEGF and IGF-1 levels in uterine tissue extracts from each group [*P < 0.05, **P < 0.01, ***P < 0.001, ****P < 0.0001] (n = 6/group). Fig. S6. B-exos or MB-exos impairs the inhi [file 13287_2024_3846_MOESM1_ESM.zip › 13287/figure S9.pdf]
