# Supplementary material for: miR-340-3p-modified bone marrow mesenchymal stem cell-derived exosomes inhibit ferroptosis through METTL3-mediated m6A modification of HMOX1 to promote recovery of injured rat uterus
Source: Stem Cell Res Ther. 2024 Jul 29;15:224. doi: 10.1186/s13287-024-03846-6 (PMC11287883; doi:10.1186/s13287-024-03846-6)
Supplement: Supplementary file 2 — Additional file 2. [file 13287_2024_3846_MOESM2_ESM.docx]

Table S1 Primer Sequences used for RT-qPCR

| Genes | Forward primer | Reverse primer |
| --- | --- | --- |
| Rat METTL3 | 5′-CTTTAGCATCTGGTCTGGGCT-3′ | 5′-CCTTCTTGCTCTGCTGTTCCT-3′ |
| Rat WTAP | 5′-GCAAGAGTGCACCACTCAAA-3′ | 5′-CATTTTGGGCTTGTTCCAGT-3′ |
| Rat KIAA1429 | 5′-CCTGGAAAGTCACCCAGTGT-3′ | 5′-GGTCCGAACTTGAAGCAAAG-3′ |
| Rat METTL14 | 5′-TCCTCCCAAATCCAAGTCTG-3′ | 5′-ACCTCTGTGTGTCCCTCCAC-3′ |
| Rat FTO | 5′-TCTTCACCAGGGAGACTGCT-3′ | 5′-CAACTGGCAGCGTTGTAAGA-3′ |
| Rat ALKBH5 | 5′-CTCAGTGGGTATGCTGCTGA-3′ | 5′-GGGTCTCTGGTGTTTCCTGA-3′ |
| Rat YTHDF1 | 5′-CCCTGTCCTGGAGAAACTGAAAGC-3' | 5′-GTACTTGATGGAGCGGTGGATGTC-3' |
| Rat YTHDF2 | 5′-TGCCATGTCAGACTCCTACTTACCC-3' | 5′-TCTCCGTTGCTCAGTTGTCCATAAG-3' |
| Rat YTHDF3 | 5′-GCAGTTACGGCTATCCACCTAGTTC-3' | 5′-AGTCCAGTCATGCCTTGCTCAATAC-3' |
| Rat YTHDC1 | 5′-ACAGGGCAACAACACTGAGAATGAG-3' | 5′-CCTCCTCCTCCTCCGCATCTTC-3' |
| Rat YTHDC2 | 5′-GCAACAGACCACCCTCACAGAATG-3' | 5′-GAAGACAGCATCACCTCCATCATCC-3' |
| Rat HNRNPC | 5′-GCTGGAGAGGATGGCAGAATGATTG-3' | 5′-GAGGGGACGGAGAAGGGTGTTC-3' |
| Rat HNRNPA2B1 | 5′-GAGGAGGATATGGTGGTGGAGGAC-3' | 5′-TTGGACCGTAGTTAGAAGGTTGCTG-3' |
| Rat Col1α1 | 5′-GAGAGCATGACCGATGGATT-3' | 5′-TTGAGGTTGCCAGTCTGTTG-3' |
| Rat Fn1 | 5′-GCGACTCTGACTGGCCTTAC-3' | 5′-CCGTGTAAGGGTCAAAGCAT-3' |
| Rat α-SMA | 5′-TTCAATGTCCCTGCCATGTA-3' | 5′-GAAGGAATAGCCACGCTCAG-3' |
| Rat GAPDH | 5′-GACATGCCGCCTGGAGAAAC-3′ | 5′-AGCCCAGGATGCCCTTTAGT-3′ |

Table S2. Primer Sequences used for MeRIP-qPCR

| Genes | Forward primer | Reverse primer |
| --- | --- | --- |
| Rat FTH1 | 5'-GCTGAATGCAATGGAGTGTG-3' | 5'-TCTTGCGTAAGTTGGTCACG-3' |
| Rat SLC7A11 | 5'-TGCCTTGTCTGCTTTGTGTC-3' | 5'-GAATTGCAGGGAACTGTGGT-3' |
| Rat HMOX1 | 5'-GAAGAAGATTGCGCAGAAGG-3' | 5'-GAAGGCGGTCTTAGCCTCTT-3' |
| Rat STAT3 | 5'-CAGCCAAACTCCCAGATCAT-3' | 5'-TCTGCTTTCACAGCCATCAC-3' |
| Rat PLA2G6 | 5'-GTCAGTGATGCAGTGCTGGT-3' | 5'-CCGAGCCCTAGTCTCAGATG-3' |
| Rat FXN | 5'-CTATGACTGGACCGGGAAGA-3' | 5'-CTGGGGTCTTTGCCTGATAG-3' |
| Rat SCD | 5'-GGCTTCCAGATCCTCCCTAC-3' | 5'-CAACAACCAACCCTCTCGTT-3' |
| Rat CDH1 | 5'-CTATGCTGGGTGTGTTGGTG-3' | 5'-TCCCTTTTCCCATCAGAGTG-3' |
