# Supplementary material for: miR-340-3p-modified bone marrow mesenchymal stem cell-derived exosomes inhibit ferroptosis through METTL3-mediated m6A modification of HMOX1 to promote recovery of injured rat uterus
Source: Stem Cell Res Ther. 2024 Jul 29;15:224. doi: 10.1186/s13287-024-03846-6 (PMC11287883; doi:10.1186/s13287-024-03846-6)
Supplement: Supplementary file 3 — Additional file 3. [file 13287_2024_3846_MOESM3_ESM.pdf]

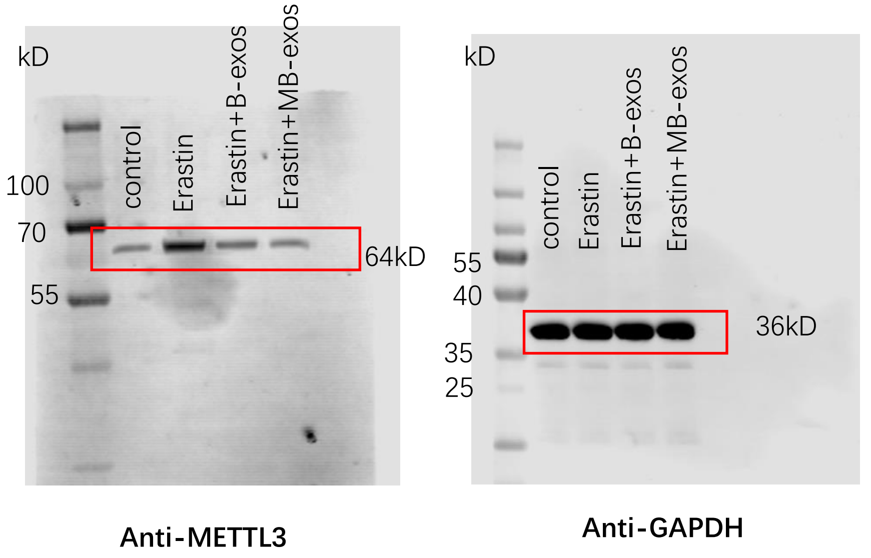

Figure 4E

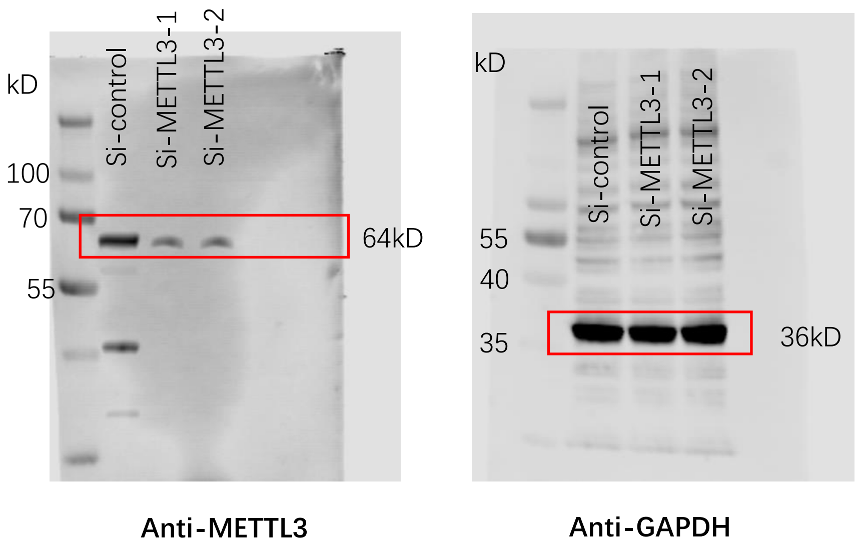

Figure 4H

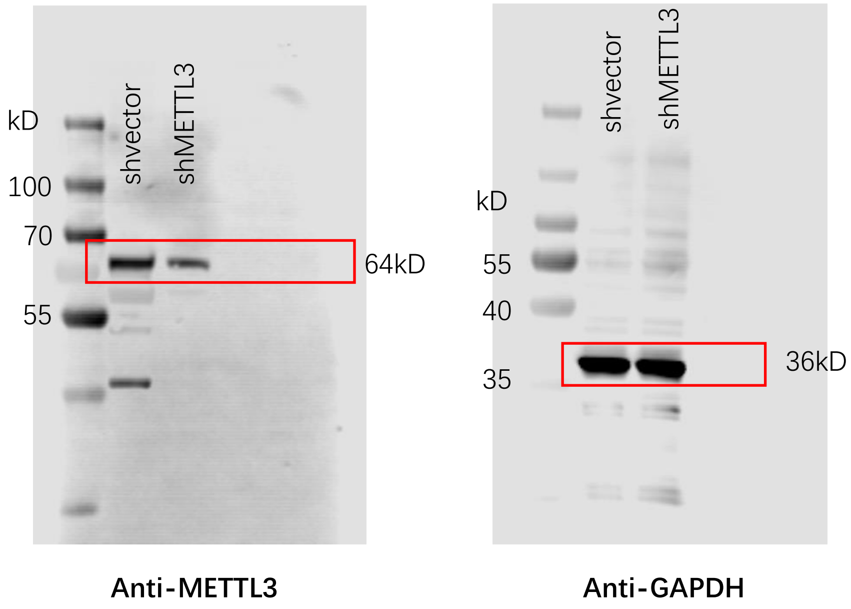

Figure 5A

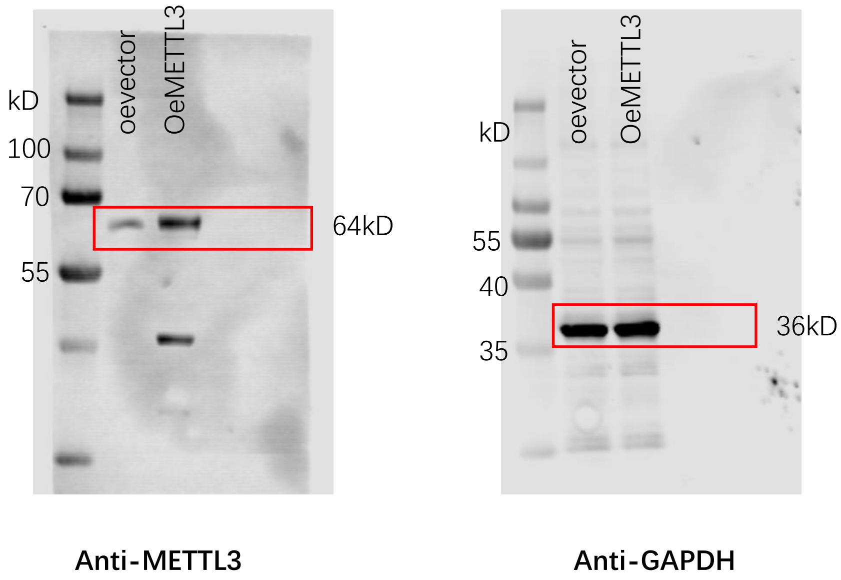

Figure 5I

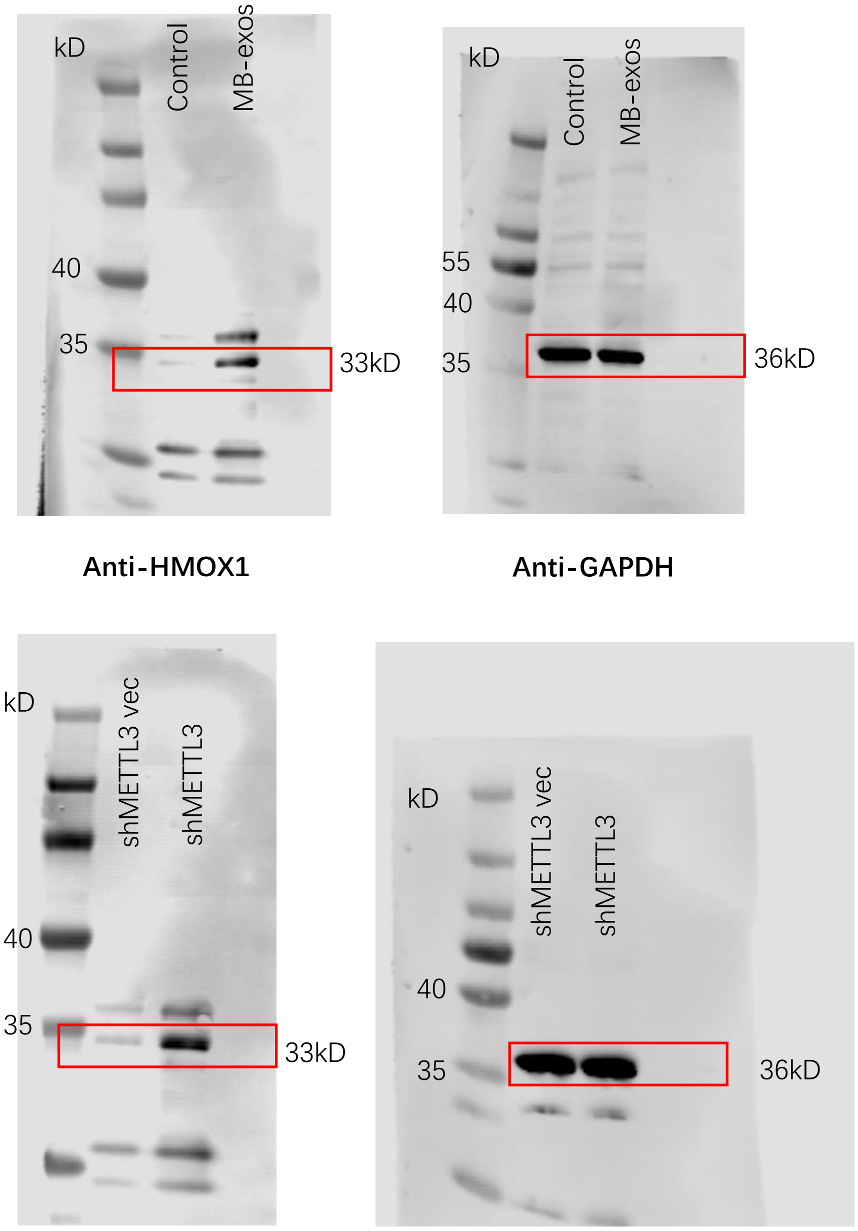

Anti-HMOX1 **Anti-GAPDH**

Anti-HMOX1 **Anti-GAPDH**

Figure6D

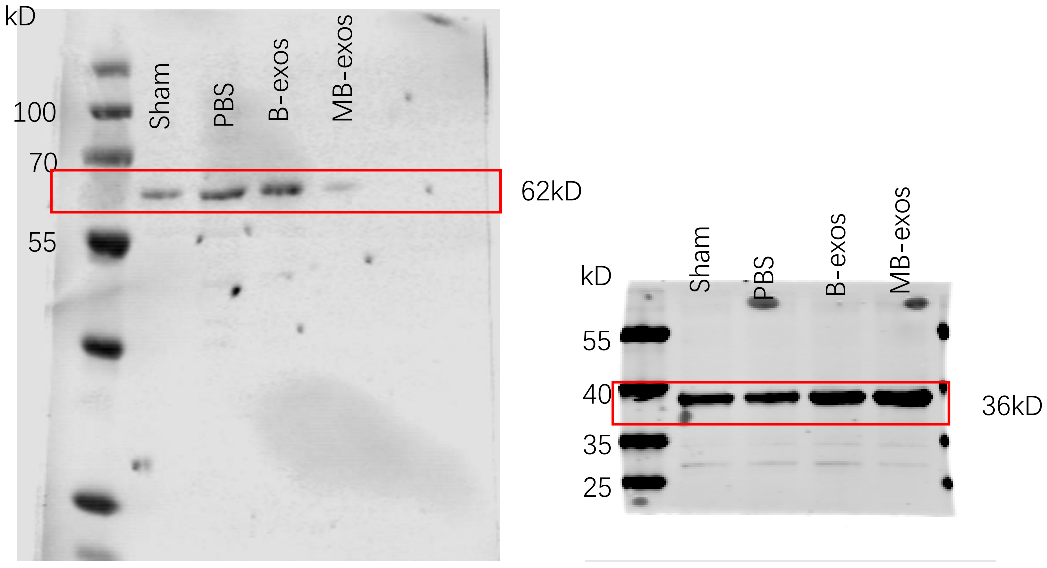

Anti-YTHDF2 **Anti-GAPDH**

Figure7B

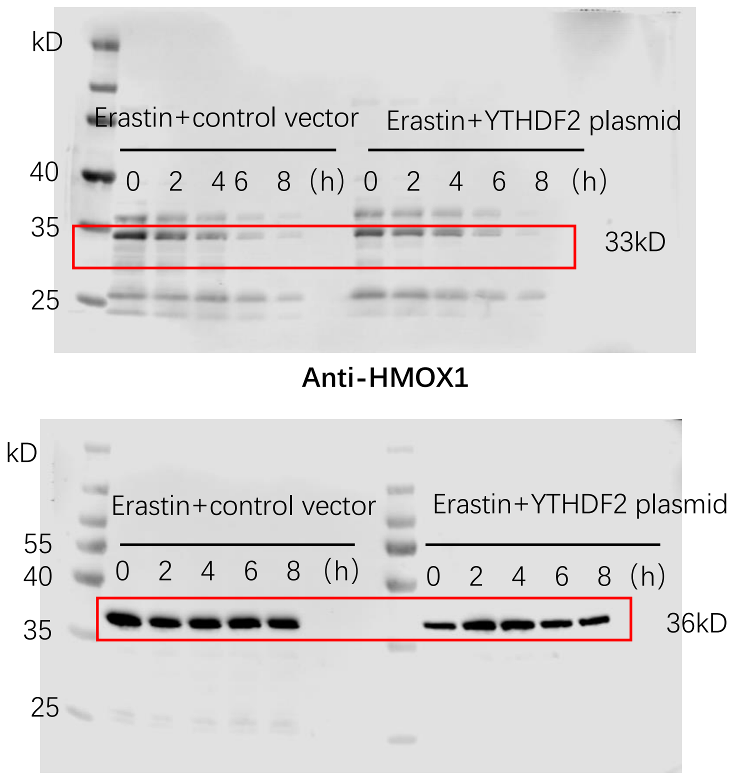

Anti-GAPDH

Figure7E

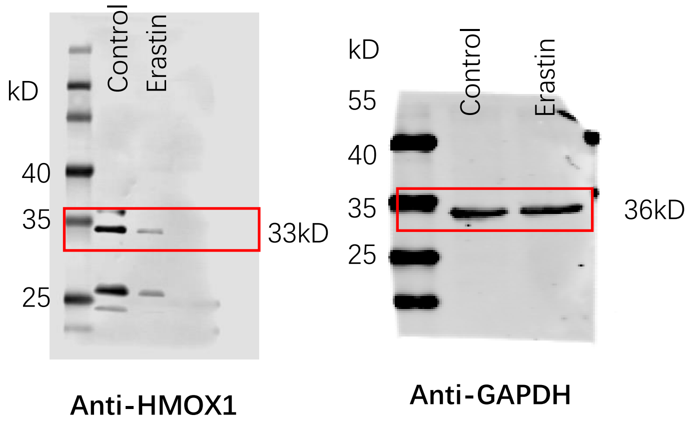

HMOX1-3’UTR-WT

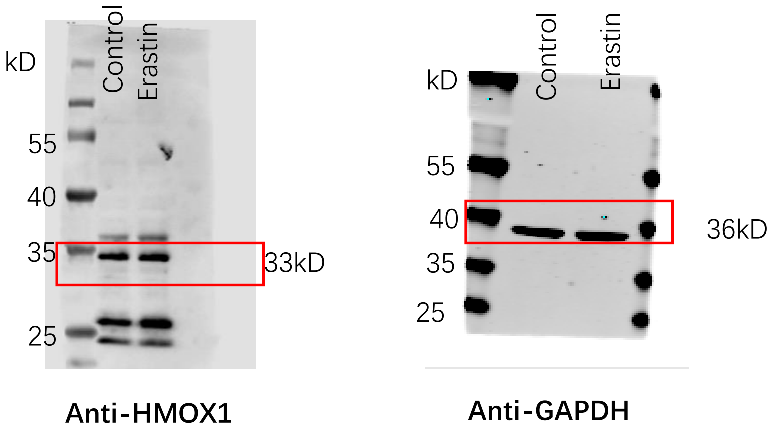

HMOX1-3’UTR -mut1

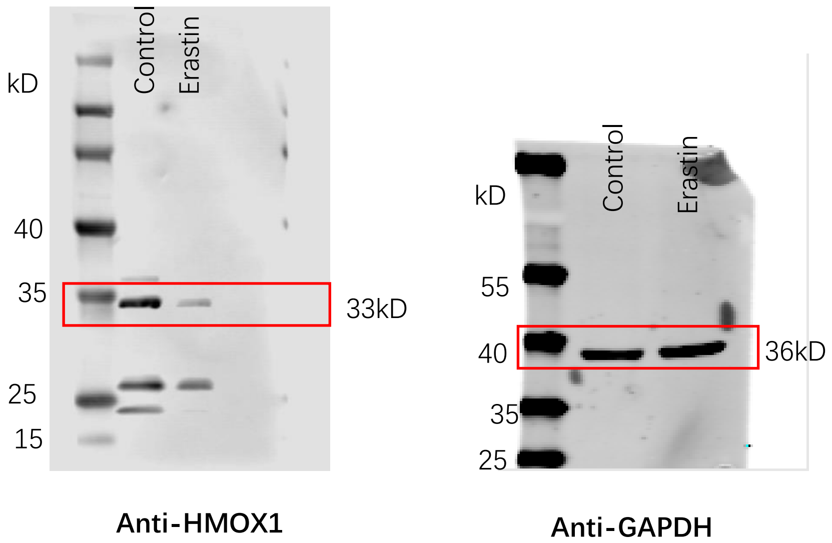

HMOX1-3’UTR -mut2

Figure7L

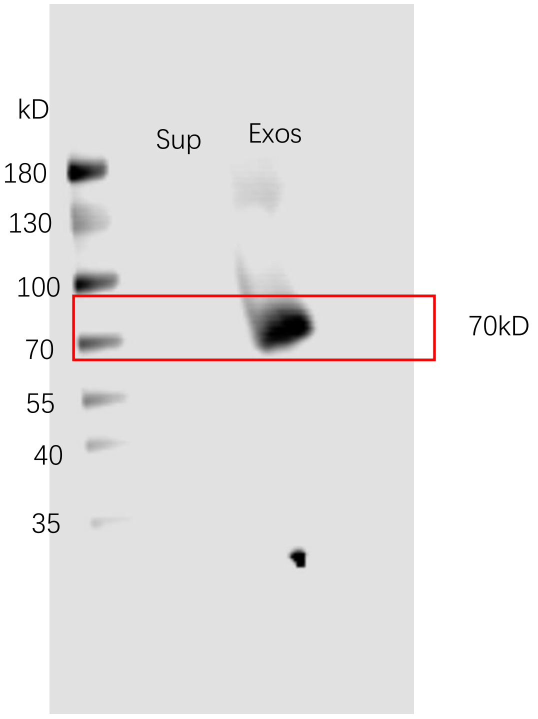
kD
Sup
Exos
180
130
100
70
55
40
35
70kD

HSP70

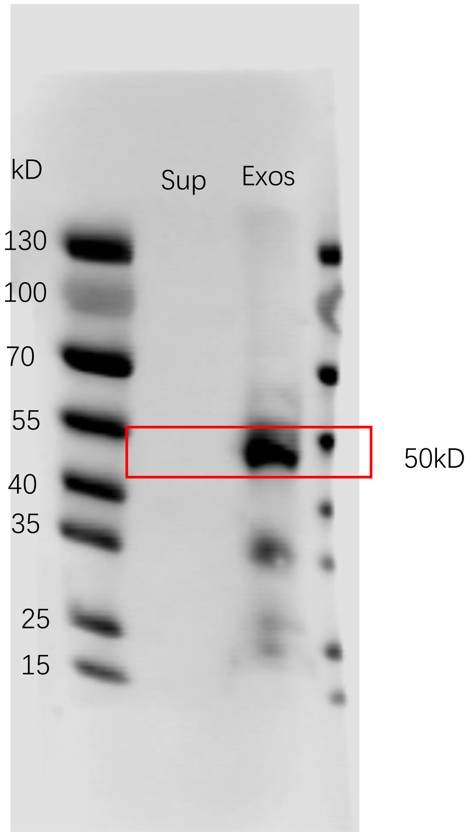
kD
Sup
Exos
130
100
70
55
40
35
25
15
50kD

CD63

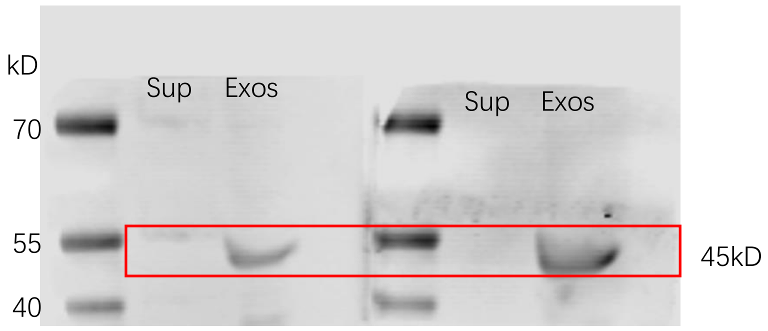
kD
Sup
Exos
Sup
Exos
70
55
40
45kD

TSG101

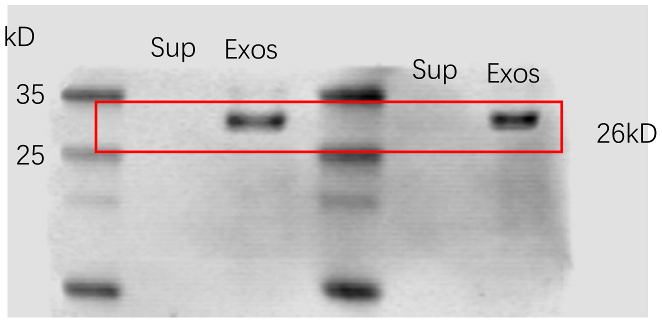
kD
Sup
Exos
Sup
Exos
35
25
26kD

CD81

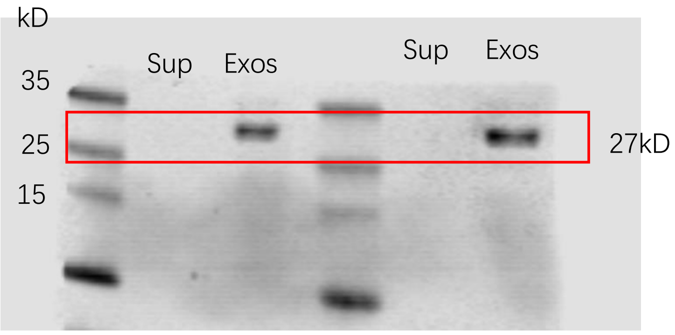

CD9

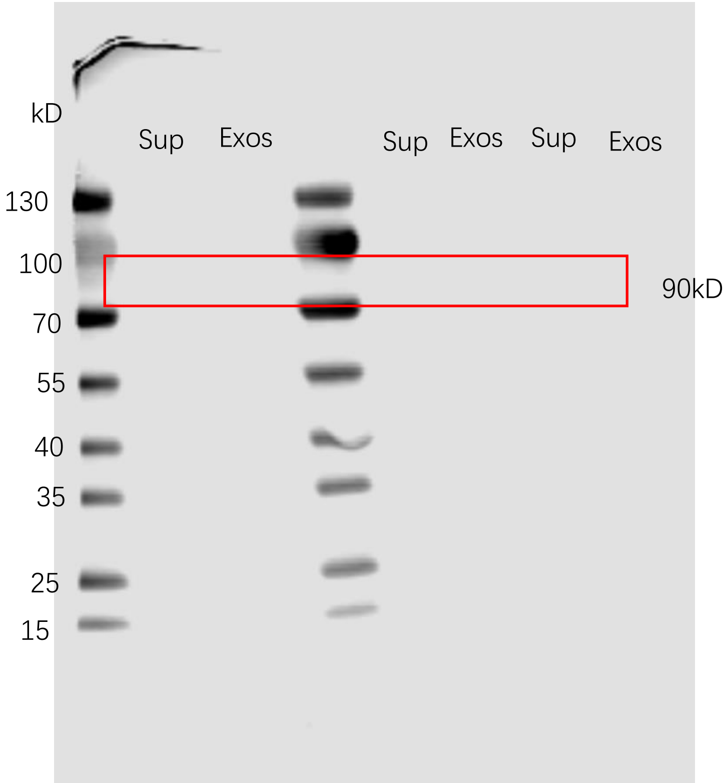

Calnexin

Figure S2
